# Molecular Characterization and Genome Mechanical Features of Two Newly Isolated Polyvalent Bacteriophages Infecting *Pseudomonas syringae* pv. *garcae*

**DOI:** 10.3390/genes15010113

**Published:** 2024-01-18

**Authors:** Erica C. Silva, Carlos A. Quinde, Basilio Cieza, Aakash Basu, Marta M. D. C. Vila, Victor M. Balcão

**Affiliations:** 1 VBlab—Laboratory of Bacterial Viruses, University of Sorocaba, Sorocaba 18023-000, SP, Brazil; erica.silva@edu.uniso.br (E.C.S.); marta.vila@prof.uniso.br (M.M.D.C.V.); 2 Department of Biological Sciences, University of South Carolina, Columbia, SC 29208, USA; amadeoalfaroquinde@gmail.com; 3 Department of Biophysics and Biophysical Chemistry, Johns Hopkins University, Baltimore, MD 21218, USA; bciezah1@gmail.com; 4 Department of Biosciences, Durham University, Durham DH1 3LE, UK; aakash.basu@durham.ac.uk; 5 Department of Biology and CESAM, University of Aveiro, Campus Universitário de Santiago, P-3810-193 Aveiro, Portugal

**Keywords:** bacteriophage, *Pseudomonas syringae* pv. *garcae*, coffee bacterial blight, genomic structural features, *Tequatrovirus* and *Phapecoctavirus* genuses, adsorption features, virion morphogenesis yield

## Abstract

Coffee plants have been targeted by a devastating bacterial disease, a condition known as bacterial blight, caused by the phytopathogen *Pseudomonas syringae* pv. *garcae* (Psg). Conventional treatments of coffee plantations affected by the disease involve frequent spraying with copper- and kasugamycin-derived compounds, but they are both highly toxic to the environment and stimulate the appearance of bacterial resistance. Herein, we report the molecular characterization and mechanical features of the genome of two newly isolated (putative polyvalent) lytic phages for Psg. The isolated phages belong to class Caudoviricetes and present a myovirus-like morphotype belonging to the genuses *Tequatrovirus* (PsgM02F) and *Phapecoctavirus* (PsgM04F) of the subfamilies *Straboviridae* (PsgM02F) and *Stephanstirmvirinae* (PsgM04F), according to recent bacterial viruses’ taxonomy, based on their complete genome sequences. The 165,282 bp (PsgM02F) and 151,205 bp (PsgM04F) genomes do not feature any lysogenic-related (integrase) genes and, hence, can safely be assumed to follow a lytic lifestyle. While phage PsgM02F produced a morphogenesis yield of 124 virions per host cell, phage PsgM04F produced only 12 virions per host cell, indicating that they replicate well in Psg with a 50 min latency period. Genome mechanical analyses established a relationship between genome bendability and virion morphogenesis yield within infected host cells.

## 1. Introduction

Plants affected by emerging phytopathogens could sustain huge annual global harvest losses [1]. Plant pathogens reduce both the yield and quality of agricultural production, leading to substantial economic losses [1], with plant disease outbreaks posing significant risks to global food safety and planetary environmental sustainability [2,3,4,5,6]. While plant infections by phytopathogens are currently tackled with kasugamycin and/or copper-based products, copper is harmful to the environment and has the potential to promote bacterial resistance to this metal [7,8,9,10]; on the other hand, the extensive use of kasugamycin can also promote phytopathogen resistance to this antibiotic [11].

The phytopathogenic bacterium *Pseudomonas syringae* pv. *garcae* (Psg) is responsible for the coffee halo blight disease of coffee plants [12,13,14,15,16,17,18].

One of the most promising alternatives to using antibiotics and/or copper-based products for controlling Psg lies in the use of bacterial viruses (i.e., bacteriophages or phages) as a more targeted approach, aiming at killing specifically this phytopathogen. Over the past decade, several researchers have attempted to use phages to control phytopathogens [8,9,10,12,19,20,21,22,23,24,25,26], either in vitro or ex vivo. Still, to the best of our knowledge, no phage-based strategy has been developed to try to control Psg and halt the bacterial blight of coffee. Hence, knowing in more detail the structural and molecular features of Psg lytic phages as potential silver bullets for killing such phytopathogen is a step forward in the path to developing environmentally friendly alternatives for its biocontrol.

Several researchers have reported that the dsDNA molecule can be pretty flexible on short base pair (bp) lengths, hinting that dsDNA is notably soft on 50–100 bp lengths [27,28,29]. While the dsDNA molecule’s intrinsic cyclizability (or bendability) is a central key for a myriad of essential cellular mechanisms [30,31,32], the bp sequence may have a deep impact on its mechanical features [27,30,33]. According to Basu et al. [27], a short DNA sequence abundant in A/T dinucleotides separated by 5-bp sequences from a short sequence abundant in G/C dinucleotides, along with periodicities in A/T dinucleotides at the helical repeat, endows a high cyclizability to the DNA molecule. The mechanical features of dsDNA might also impact its transcription since contacts between enhancer and promoter in the prokaryote genome are very sensitive to its mechanical properties [27]. While a rigid dsDNA structure has been associated with an uncommonly low intrinsic cyclizability [34], the side of the dsDNA with abundant T/A exhibits a significantly higher intrinsic cyclizability, being consistent with the rationale that the RNA-polymerase interacts and negotiates better with a dsDNA sequence that is more bendable (i.e., with a higher intrinsic cyclizability) [27,30]. The idea embodied here is an uncommon and highly creative approach, bringing together the mechanical features of the phage genome and predictions for its intrinsic cyclizability, aiming at unveiling a putative correlation with virion morphogenesis yield within infected bacterial host cells.

In the research work entertained herein, we report the complete molecular characterization of two previously isolated (potentially polyvalent) bacteriophages infecting Psg. Deeper analyses of the mechanical properties of the two Psg lytic phage genomes were also undertaken, in addition to full genome cyclizability calculations. Full genomic analyses showed that phages PsgM02F and PsgM04F presented a myovirus-like morphotype, belonging to the genuses *Tequatrovirus* (PsgM02F) and *Phapecoctavirus* (PsgM04F) of the subfamilies *Straboviridae* (PsgM02F) and *Stephanstirmvirinae* (PsgM04F).

## 2. Materials and Methods

### 2.1. Biological Material

The phages described in this work (phages PsgM02F and PsgM04F) were isolated from samples of coffee plant leaves (seven coffee plant leaves (average individual weight: 0.6919 ± 0.1296 g; total weight: 4.8434 g) with yellow blight disease symptoms) collected in Itu city, Brazil, viz. at Fazenda Santo António da Bela Vista (Itu, SP, Brazil) [23°18′41.1″ S, 47°24′13.2″ W] on 16 September 2022), using the collection strain Psg IBSBF-158 as host and the enrichment method described in detail elsewhere [12,25,33,35,36,37], with modifications, and thoroughly characterized from physicochemical and biological points of view in a previous publication [12]. The host strain was obtained from the Phytobacteria Culture Collection of Instituto Biológico (IBSBF, Campinas, SP, Brazil).

### 2.2. Chemicals

The magnesium sulfate was from Labsynth (Diadema, SP, Brazil). Tryptic Soy Agar (TSA) and Tryptic Soy Broth (TSB) culture media were from Sigma-Aldrich Brazil (Cotia, SP, Brazil). Bacteriologic solid agar was from Gibco Diagnostics (Madison, WI, USA). Sterilizing filtration systems Stericup™-GP (with 0.22 µm pore diameter polyethersulphate membrane) were purchased from Merck-Millipore (Darmstadt, Germany). The ultrapure water utilized had a resistivity of 18.18 MΩ.cm and conductivity of 0.05 µS.cm^−1^.

### 2.3. Phage PEG-Precipitation

Fifty milliliters of phage suspension (10^11^ PFU/mL) was added with a sterile mixture of polyethylene glycol (PEG) 8000 (Sigma-Aldrich, St. Louis, MO, USA) (10%, *w*/*w*) and NaCl (1 M) (Sigma-Aldrich), in a volumetric proportion of 2:1, respectively. The resulting suspension was incubated overnight at 4 °C and then centrifuged at 11,000 rpm (4 °C, 45 min). The supernatant was discarded, and the pellet was resuspended and homogenized in 5 mM MgSO_4_ (Sigma-Aldrich, St. Louis, MO, USA).

### 2.4. Transmission Electron Microscopy (TEM) Analyses

Before the negative staining procedure, a small volume of PEG-concentrated phage suspension (prepared as described above) was centrifuged (4 °C, 150 min, 45,000 rpm, 124,740× *g*) in a benchtop Beckman–Coulter ultracentrifuge (model Optima TLX micro-ultracentrifuge) with a TLA-55 Fixed-Angle Rotor (Indianapolis, IN, USA). The pellet was carefully collected and negatively stained with uranyl acetate (Sigma-Aldrich, St. Louis MO, USA) at 2% (*w*/*v*) as described elsewhere [12,35,36]. The electron microscopy analyses were performed in a Transmission Electron Microscope from JEOL (model JEM 2100, Tokyo, Japan), encompassing a LaB_6_ filament, operating at 200 kV and with resolution of 0.23 nm; a high-resolution CCD camera from GATAN Inc. (model ORIUS™ 832.J4850 SC1000B, Pleasanton, CA, USA) with a resolution of 11 Mp (4.0 × 2.7 k pixels/9 × 9 µm^2^) was utilized for the acquisition of digital images, via software Gatan Microscopy Suite (DigitalMicrograph from Gatan Inc., version 2.11.1404.0, Pleasanton, CA, USA). To determine the average size of virion capsid and tail, seven phage particles were measured using the public domain ImageJ software (version 1.52a) from the National Institute of Health (NIH; Bethesda, MD, USA).

### 2.5. Phage Virion Whole Genome Sequencing

Purified DNA samples of the isolated phages were sequenced at NGS SOLUÇÕES GENÔMICAS (Piracicaba, SP, Brazil) using the Illumina MiSeq platform. PEG-concentrated phage suspensions (500 µL) were treated with 1.25 µL DNase-I (20 mg /mL, TransGen Biotech, Beijing, China) and 1.25 µL RNase (10 mg/mL, TransGen Biotech, Beijing, China) at 37 °C for 1 h. Following incubation, 1.25 µL proteinase K (TransGen Biotech, 20 mg/mL), 25 µL aqueous SDS (Sigma-Aldrich, St. Louis, MO, USA) (10%, *w*/*w*) (final SDS concentration of 0.5%, *w*/*w*) and 20 µL aqueous EDTA (Sigma-Aldrich, St. Louis, MO, USA) (0.5 M, pH 8.0) (final EDTA concentration of 20 mM) were added to samples, followed by incubation at 60 °C for 1 h, after which the mixture was allowed to cool down to room temperature. DNA extraction was performed using the phenol: chloroform (Sigma-Aldrich, St. Louis, MO, USA) protocol as briefly described. Phenol was added in a 1:1 (*v*/*v*) proportion. After centrifugation at 6000 rpm for 5 min, an equal volume of chloroform was added to the supernatant, and this last step was repeated twice. After centrifugation (6000 rpm for 5 min), the aqueous phase was carefully collected. DNA was precipitated by adding the supernatant to 1/10 volume 3 M NaOAc (Sigma-Aldrich, St. Louis, MO, USA) (pH 7.5) and 2.5× volume of cold absolute ethanol (Sigma-Aldrich, St. Louis, MO, USA). The resulting mixture was incubated overnight at −20 °C and −86 °C for 30 min and then centrifuged at 14,000 rpm for 20 min. The supernatant was discarded, and the pellet was allowed to dry, after which it was dissolved in 50 µL of nuclease-free ultrapure water (Thermo Scientific, Waltham, MA, USA). Purified phage DNA was subjected to a final clean-up step using RNase and stored at −20 °C. DNA purity and concentration were evaluated on a DS-11FX spectrophotometer (DeNovix Inc., Wilmington, DE, USA) at 260 nm, 280 nm and 230 nm. Further quantification was performed with Quant-iT Picogreen dsDNA assay kit (Life Technologies, Carlsbad, CA, USA). DNA integrity was examined with a DNA 7500 chip using a 2100 Bioanalyzer (Agilent, Palo Alto, CA, USA).

Purified phage DNA (2–20 ng) was used to prepare the shotgun genomic library with the Illumina Nextera DNA library preparation kit (Illumina, San Diego, CA, USA). The DNA fragment library was cleaned up with Agencourt AMPure XP beads (Beckman Coulter, Indianapolis, IN, USA), and the average fragment size (400–700 bp) was verified by running in the 2100 Bioanalyzer using Agilent High Sensitivity DNA chip (Agilent, Palo Alto, CA, USA). The quantification of the Illumina sequencing library via quantitative PCR, clusterization, normalization, and sequencing was performed following standard protocols for sequencing in the Illumina MiSeq platform. The library was subjected to one run using the MiSeq Reagent kit v3 (600-cycle format, paired-end (PE) reads).

### 2.6. Phage Virion Genome Assembly, Taxonomic Evaluation, Annotation, and Phylogeny

*Evaluation of sequencing reads.* The sequencing data obtained by NextSeq 2000 (Illumina, Inc., San Diego, CA, U.S.A.) were analyzed by the software BCL Convert version 3.8.4, which base calls the sequencing images, converting them into sequences in fastq format, with each base accompanied by a Phred quality score [38]. Sequencing quality was evaluated with FastQC version 0.12.1 (https://www.bioinformatics.babraham.ac.uk/projects/fastqc/ (accessed on 14 August 2023)), and a unified report was obtained with MultiQC version 1.14 [39].

*Phage virion genome assembly*. The sequencing reads were processed, and the genome was assembled using Shovill 1.1.0 (https://github.com/tseemann/shovill (accessed on 15 August 2023)), a pipeline specialized in assembling prokaryotic genomes. In order to achieve a fast and accurate assembly, Shovill performs several steps using various bioinformatics tools: (1) Estimates genome size using KMC version 3.2.1 [40]; (2) Reduces the number of sequencing reads to get around 250× coverage over estimated genome size; (3) Remove Illumina adapters with Trimmomatic version 0.39 [41]; (4) Fixes bugs in sequencing reads using Lighter version 1.1.2 [42]; (5) Joins paired readings that overlap, with FLASH version 1.2.11 [43]; (6) Assembles the draft genome with SPAdes version 3.15.5 [44]; (7) Maps sequencing reads into the draft genome assembled with BWA MEM version 0.7.17 [45]; (8) With the mapping obtained in the previous step, corrects inaccuracies in the assembly using Pilon version 1.24 [46]; and (9) Removes very short contigs (minimum size of 500 base pairs), either with very low coverage or formed exclusively by homopolymers. After assembly, the sequencing reads were mapped onto the assembly contigs using Minimap2 version 2.25 [47] to estimate the sequencing coverage of each contig.

*Taxonomic evaluation*. The taxonomic evaluation of contigs was performed with Kraken2 version 2.1.2 [48], a taxonomic classifier based on kmers, using the PlusPF database version 20230605 (available at https://benlangmead.github.io/aws-indexes/k2 (accessed on 18 August 2023)). The viral contigs were separated according to the genus identified by Kraken2 and KrakenTools version 1.2 [49]. Then Ccfind version 1.4.5 [50] was used to identify circular contigs and remove terminal repeats from circular contigs. Finally, BLAST+ version 2.14.0 [51] was used to determine the bacteriophage most similar to the retrieved viral contigs.

*Phage virion genome annotation*. The genome was annotated with the Pharokka annotation pipeline version 1.3.2 [52] and Pharokka Database v1.4.0 (https://zenodo.org/records/8267900 (accessed on 21 August 2023)). Pharokka is a specialized pipeline for the annotation of phage genomes that combines several tools to make the prediction and obtain a functional annotation of several categories of genes: (1) Transfer RNAs and transfer-messenger RNAs, with tRNAscanSE version 2.0.12 [53] and ARAGORN version 1.2.41 [54]; (2) Clusters of CRISPRs, with MinCED version 0.4.2. MinCED is a reimplementation of the program CRT [55]; (3) Protein-coding genes are predicted with PHANOTATE version 1.5.1 [56] and annotated with similarity searches using Mmseqs2 version 13.45111 [57] against custom versions of the databases PHROG [58], CARD [59] and VFDB [60]. Circular maps of the annotated phage genomes were generated using Pharokka (version 1.3.2) [52].

*Lineage phylogeny and taxonomy*. Initially, ReferenceSeeker version 1.8.0 [61] was used to identify the *Tequatrovirus* genomes most similar to the genome assembled for phage PsgM02F. ReferenceSeeker estimates the similarity between genomes based on genomic sketches calculated with Mash version 2.3 [62] and average nucleotide identity (ANI) calculated from alignments made with the nucmer program from the MUMmer package version 4.0.0-RC1 [63]. ReferenceSeeker uses a database based on RefSeq/NCBI bacterial genomes [64]. The genomes identified by ReferenceSeeker were used to develop a phylogenetic hypothesis using Mashtree version 1.2.2 [65], which estimates distances between genomes by constructing approximate local alignments. Nine hundred and ninety-nine bootstraps were performed, and QuickTree version 2.5.0 [66] was used to build the phylogenetic tree (Phage PsgM02F) by the neighbor-joining method. Regarding the taxonomy of phage PsgM04F, vConTACT2 [67] alongside the complete Millard Lab phage genome database (~21,000 genomes) [68] (version of 5 January 2023) was used. Phages PsgM02F and PsgM04F were classified into Family when directly connected on the network to a family group and at genus level when directly clustered. Following that analysis, the protein cluster file generated by vConTACT2 [67] was used to identify core proteins of all phages classified as being from the same family. All core proteins were retrieved, aligned with MAFFT [69], and concatenated. The alignment was then used as input for FastTree [70] for Maximum Likelihood phylogenetic calculation with the Whelan–Goldman model [71] followed by γ optimization [72]. The resulting tree (phage PsgM04F) was visualized using the FigTree (https://github.com/rambaut/figtree/releases/tag/v1.4.4 (accessed on 25 August 2023)) program.

### 2.7. Mechanical Properties of the PsgM02F and PsgM04F Phage Genomes

*DNA structural features.* DNA is sequence-dependent, and its study is important in genome-wide analysis. Finding out the structural features of DNA can help reveal the preferred conformations that are intrinsic to a given DNA sequence and its dynamics. To do that, we used “DNAshape”, a web-based application that uses Monte Carlo simulations in high-throughput (HT) studies that can predict multiple DNA structural features such as Minor Grove Width (MGW), Roll, Propeller twist (ProT) and Helix twist (HelT). DNA shape features at a single-nucleotide position are determined by the sequence context of the corresponding bp. The context is the immediate neighbors of a bp or a larger number of adjacent bp, which in turn is characterized as a function environment of its pentameric environment. In summary, each one of the features was determined entirely by the nucleotide sequence context of the genomes, using a high-throughput methodology that includes a pentamer model to predict the structural values except for the two terminal bp in MGW and ProT, or one bp step at each end in roll and HelT [73]. The DNA structural features of the genomes of the two phages were calculated according to the procedures described in detail by Harada et al. [33] and Balcão et al. [30] using the “DNAshape” web-based application (http://rohslab.cmb.usc.edu/DNAshape/ (accessed on 4 September 2023)) [73]. A Python (version 3.9.12) custom script for plotting the resulting heatmaps data was then created and run in Jupyter Notebook (version 6.4.8) within Anaconda Navigator (version 2.1.4, Anaconda Inc., Austin, TX, USA). Once the predicted values were obtained, data was fully analyzed, and its characteristics were obtained to better understand the predicted values. Correlations between the four structural features were then analyzed, and heatmaps plotting the number of nucleotides per genome and the four structural features were produced.

*Correlation of the DNA shape of both genomes.* Once the values were predicted, a pairwise correlation of the DNA shape was computed to quantify their linear relationship using a custom Python script.

*Dinucleotide distance correlation patterns of both PsgM02F and PsgM04F genomes.* In this analysis, we computed the pairwise distance distribution function following the procedures outlined by Basu et al. [74]. The pairwise distance distribution function is a measure of how frequently two specific dinucleotides occur at a given separation within a DNA sequence. This separation is quantified in terms of nucleotide intervals. We have explored the self-pairwise distance distribution function for the sixteen dinucleotide combinations possible, viz. AA, AC, AG, AT, CA, CC, CG, CT, GA, GC, GG, GT, TA, TC, TG, and TT, independently for each genome. This was accomplished by counting the occurrences of each dinucleotide in each genome and dividing it by the respective genome’s length. To identify correlation, a 1 was assigned to each dinucleotide when it was found and 0 when it was not. Next, the frequency of closeness among the dinucleotides was calculated for a total of 100 steps. These values were compared with random expected values and plotted as a function of the 100 steps and the correlation frequency of the specific dinucleotide found in both genomes. The resulting plots illustrate the pairwise correlation between both genomes for each dinucleotide within 100 steps of distance. The PsgM02 genome is depicted in red, while the PsgM04 genome is depicted in blue. The dotted line in the graph corresponds to the random expected sequence. All the calculations were independently repeated for each of the two-phage genomes. A Python (version 3.9.12) custom script was written using Jupyter Notebook (version 6.4.8) for calculating dinucleotide distance correlations and running in Anaconda Navigator (version 2.1.4, Anaconda Inc., Austin, TX, USA).

*Frequency of the 16 dinucleotides in the PsgM02F and PsgM04F phage genomes.* One has used a Python script to investigate the net occurrence of the 16 dinucleotide combinations in the genomes of phages PsgM02F and PsgM04F. This was accomplished by counting the occurrence of each dinucleotide and dividing it by the respective genome’s length.

*Differential dinucleotide frequency between PsgM02F and PsgM04F phage genomes.* We computed the differential dinucleotide frequency between the PsgM02F and PsgM04F genome. To do that, the frequency of occurrence of each possible dinucleotide combination per genome was calculated, and then the calculated frequencies of PsgM02F were subtracted from those of PsgM04F, yielding a total of 256 differential values. The resulting data was represented as a heatmap, where a positive differential frequency is depicted in red while a negative differential frequency is depicted in blue.

*Phage genome cyclizability*. In this analysis, we calculated the cyclizability values associated with each genome following the procedure described by Basu et al. [74]. A Python (version 3.9.12) custom script for calculating genome cyclizability was created and run in Jupyter Notebook (version 6.4.8) within Anaconda Navigator (version 2.1.4, Anaconda Inc., Austin, TX, USA). Cyclizability of a genome sequence may be defined as the natural logarithm ratio of probabilities for finding sequences in the looped vs. control groups (i.e., the natural logarithm of the ratio of the relative population of a nucleotide sequence in a sample pool to that in control), whereas intrinsic cyclizability is defined as the mean over such variation, and can be regarded as a proxy for bendability [34,75]. Cyclizability values were only calculated every 7th base pair, aiming to check how the bendability changes around some important locations in the phage genomes and to simply average bendability over the entire genome and compare different phages. Cyclizability was computed using nucleotide intervals of 50 base pairs with a seven-base pair overlap for each genome. The calculations were performed independently for both PsgM02F and PsgM04F genomes. Subsequently, the results were displayed as heatmaps, and box plots were elaborated to display the statistics and distribution of cyclizability values per genome. The mean range is indicated by the black line within each box plot, and the maximum and minimum whiskers indicate the highest and lowest cyclizability values. Outliers are shown in open circles.

### 2.8. Viral Proteomic Trees

The web-based ViPTree program (https://www.genome.jp/viptree (accessed on 6 January 2024)) was used to analyze similarities and relationships between phages PsgM02F and PsgM04F and other prokaryotic dsDNA viruses. All computations were performed using the SuperComputer System of the Institute for Chemical Research of Kyoto University (Kyoto, Japan).

### 2.9. Statistical Analyses

All statistical analyses were performed using Microsoft Excel (Microsoft, Redmond, WA, USA).

## 3. Results

Two newly isolated polyvalent virulent phages preying on *Pseudomonas syringae* pv. *garcae* IBSBF-158 (Psg) cells were characterized relative to their virion morphogenesis yield within infected host cells and had their genomes sequenced, annotated, and fully analyzed from both molecular and mechanical points of view.

### 3.1. Phage Plaque Morphologies and Virion Morphotypes and Physical Features

**Figure 1:**
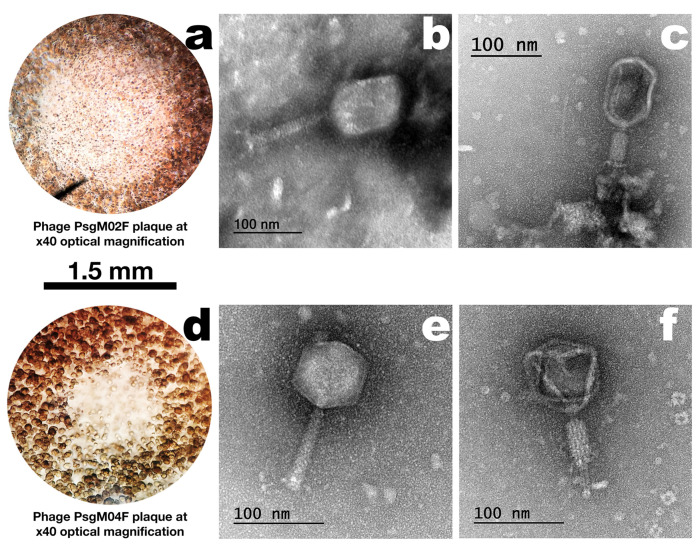
Morphology of phages PsgM02F (**a**) and PsgM04F (**d**) plaques on a lawn of their bacterial host (Psg IBSBF-158) observed under optical microscopy (40× magnification, where bacterial debris around the phage plaques are also clearly noticed), and virion morphotypes (Phage PsgM02F: (**b**,**c**); Phage PsgM04F: (**e**,**f**) obtained by TEM analysis following negative-staining). The TEM photomicrographs of the two-phage virions allow us to observe the intact head (containing the dsDNA) and uncontracted tail (Phage PsgM02F: (**b**); Phage PsgM04F: (**e**), and empty head, contracted sheath and tail tube following translocation of its dsDNA into the host) (Phage PsgM02F: (**c**); Phage PsgM04F: (**f**).

Both phages formed clear plaques on a lawn of the host (Psg IBSBF-158), with phage PsgM02F (formerly ph002F) producing larger plaques with diameters of ≈1.5 mm (Figure 1a, zoomed in plaque). Regarding phage PsgM04F (formerly ph004F), it produced tiny (≈0.5 mm, Figure 1d, zoomed-in plaque) lysis plaques on the lawn of its host. High-titre suspensions (10^11^ PFU/mL) were obtained for both phages [12].

Based on the morphological analysis of the two-phage virions by TEM (Figure 1b,c,e,f), both presented myovirus morphotypes and were identified as belonging to class Caudoviricetes. While phage PsgM02F displayed an elongated (prolate) icosahedral capsid and a long contractile tail, phage PsgM04F has a perfect icosahedral head and also a long contractile tail, with the approximate dimensions displayed in Table 1.

### 3.2. Genomic Characterization

The genomes of phages PsgM02F and PsgM04F were sequenced and duly assembled, resulting in contigs of 165,282 bp (phage PsgM02F) and 151,205 bp (phage PsgM04F). Although they could not be circularized with overlapping of both end sequences, the assembled phage genomes are complete with linear topology. The GC content of the PsgM02F phage genome is 35.4%, whereas that of the PsgM04F phage genome is 42.3%. The overall features of both genome assembly and annotation are summarized in Table 2.

**Figure 2:**
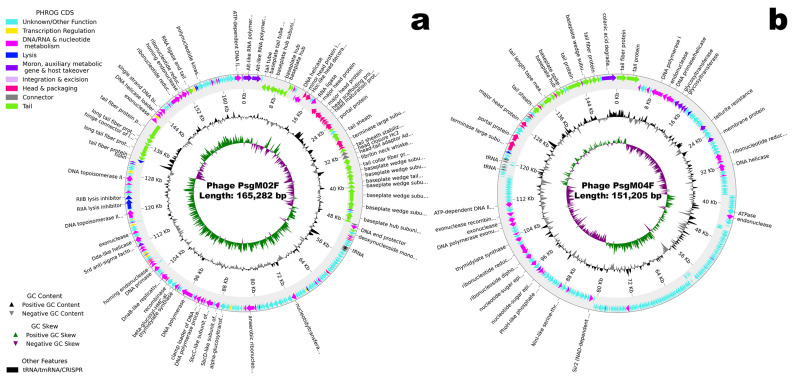
Annotated genome maps of phages PsgM02F (**a**) and PsgM04F (**b**), displaying GC skew, G + C content and predicted CDS. The colored (except light blue) arrows in the outer ring represent the annotated coding sequences (CDSs) according to the annotation in Supplementary Table S1, whereas the light blue arrows correspond to hypothetical proteins, and black arrows correspond to tRNAs. The arrows represent the direction of transcription (strand + or −).

The genome of each phage encodes 11 tRNAs, and while phage PsgM02F encodes 278 protein-coding genes (coding sequences, CDS), phage PsgM04F encodes 324 CDS (Supplementary Table S1). A comparison of the annotated CDS in the genomes of both phages with different databases unveiled that, in the genome of phage PsgM02F 142 of them are predicted as hypothetical proteins or proteins of unknown function, whereas in the genome of phage, PsgM04F 251 CDS are predicted as hypothetical proteins or proteins of unknown function. Typical structural proteins such as capsid, fibritin neck whiskers, tail, tail sheath, baseplate tail tube, baseplate, and spike proteins were annotated in phage PsgM02F genome, along with DNA metabolism-related proteins and host lysis proteins (holin, spanin). Regarding phage PsgM04F, typical structural proteins such as capsid, tail fiber, tail sheath and baseplate spike were annotated, along with DNA metabolism-related proteins and host lysis proteins (spanin, endolysin). We have not detected genes related to depolymerases, toxins, virulence factors, antibiotic resistance, or integrase enzymes among the CDS with predicted functions in both phage genomes (Supplementary Table S1). Approximately half of the protein-coding genes identified were annotated as proteins with assigned functions in the genome of phage PsgM02F (Supplementary Table S1), whereas a little less than one-quarter of the CDS identified in the genome of phage PsgM04F were annotated as proteins with assigned function (Supplementary Table S1). Circular maps of the annotated genomes of phages PsgM02F and PsgM04F are displayed in Figure 2.

**Figure 3:**
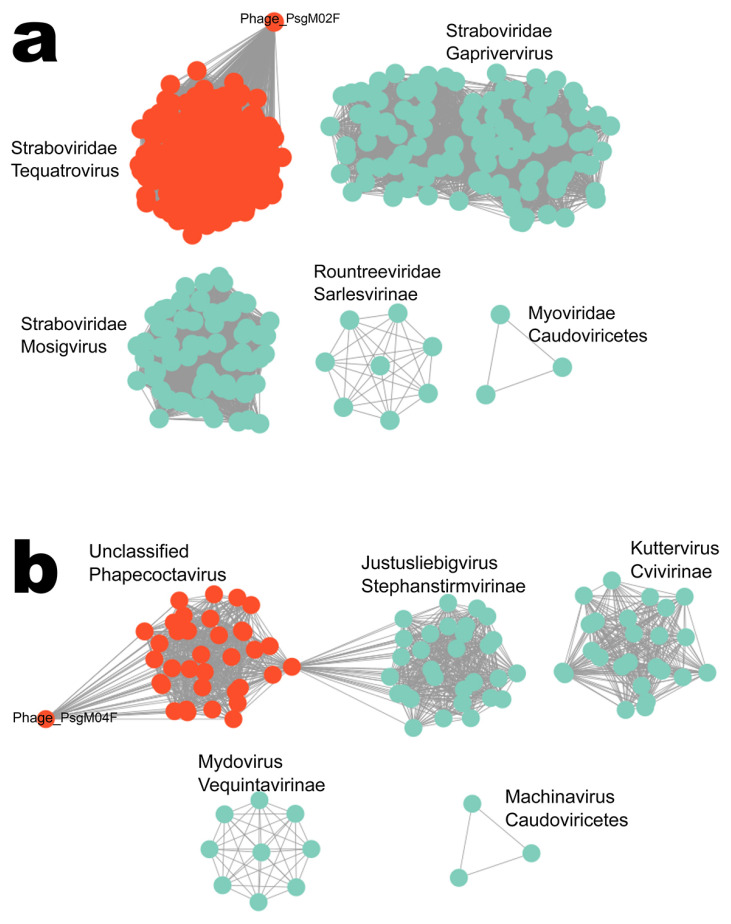
Proteome-based network analysis, calculated with vConTACT2 and visualized with Cytoskape (version 3.9.1), of phages PsgM02F (**a**) and PsgM04F (**b**). The predicted proteomes of phages PsgM02F and PsgM04F were clustered with the proteomes of their closest annotated phages pre-selected from the Millard phage database.

The proteome clustering and network analyses of phages PsgM02F and PsgM04F, calculated with vConTACT2 [67] and visualized with Cytoskape (version 3.9.1) [76], are displayed in Figure 3. Phage PsgM02F connects to one of three clusters of the family *Straboviridae*, which contains phages from the genus *Tequatrovirus* (Figure 3), whereas phage PsgM04F connects to a cluster encompassing phages from the genus *Phapecoctavirus* (Figure 3), which belongs to the subfamily *Stephanstirmvirinae* according to recent bacterial viruses’ taxonomy [77]. Interestingly, no virus isolated from Pseudomonas sp. was detected within either the *Tequatrovirus* or *Phapecoctavirus* clusters.

**Figure 4:**
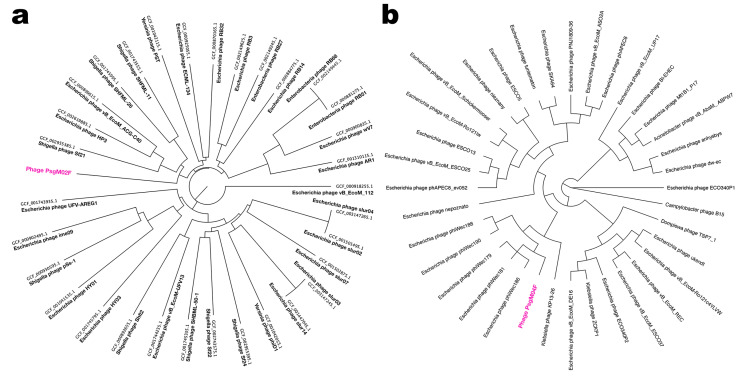
Phylogenetic trees calculated using the soft-core protein clusters from 32 phages connected with phage PsgM02F (highlighted in magenta, (**a**)) and from 35 phages connected with phage PsgM04F (highlighted in magenta, (**b**)) (data gathered from GenBank (GCA) and RefSeq (GCF) genome assemblies). Sequences of protein clusters were used for a Maximum Likelihood (ML) phylogenetic reconstruction using 1000 bootstrap replicates.

On the network analysis, phage PsgM02F was grouped with 32 phages at the family level and 5 on the same cluster (at genus level) (Figure 4a) viz. Shigella phage Sf21, Escherichia phageHP3, Escherichia phage vB_EcoM_ACG-C40, Shigella phage SHFML-26 and Shigella phage SHFML-11, whereas phage PsgM04F was grouped with 35 phages at the family level, and 6 on the same cluster (at genus level) (Figure 4b) viz. Escherichia phage phiWec179, Escherichia phage phiWec181, Escherichia phage phiWec186, Escherichia phage phiWec188, Escherichia phage phiWec190 and Klebsiella phage KP 13–26. Phylogeny was done with the core and found proteins. Here, we can see that phages PsgM02F and PsgM04F are alone on their branch, showing less phylogenetic similarity (Figure 4). The phage closest to the PsgM04F phage is Escherichia phage phiWec190 (LC739539.1). The closest genomes to the PsgM02F phage genome are GCF_002619885.1 (Escherichia phage HP3), with a Mash distance of 0.02545, and GCF_002955385.1 (Shigella phage Sf21), with a Mash distance of 0.02600 (Figure 4a). These genomes have the smallest distance to the PsgM02F phage genome, using a genomic distance metric calculated with Mash, a program that calculates distances between genomes using kmer sketches. However, this distance alone reduces the information too much. The ReferenceSeeker program calculates other similarity estimates such as ANI (average nucleotide identity) and the proportion of the conserved genome of the genome of interest in relation to the genomes in the database, and the reciprocal measures of ANI and proportion of the conserved genome of the genomes in the database against the genome of interest. Thus, the genome with the smallest Mash distance (GCF_002619885.1) is not necessarily the genome with the highest ANI (95.52%, versus 96.45% of GCF_002955385.1) or with the highest proportions of conserved genome (80.83%, versus 82.01% of GCF_002955385.1) (Supplementary Materials). According to the analyses carried out using the ReferenceSeeker program, the phages closest to the PsgM02F phage are GCF_002619885.1, with 95.52% similarity, and GCF_002955385.1, with 96.45% similarity.

### 3.3. DNA Structural Features of Phages PsgM02F and PsgM04F

**Figure 5:**
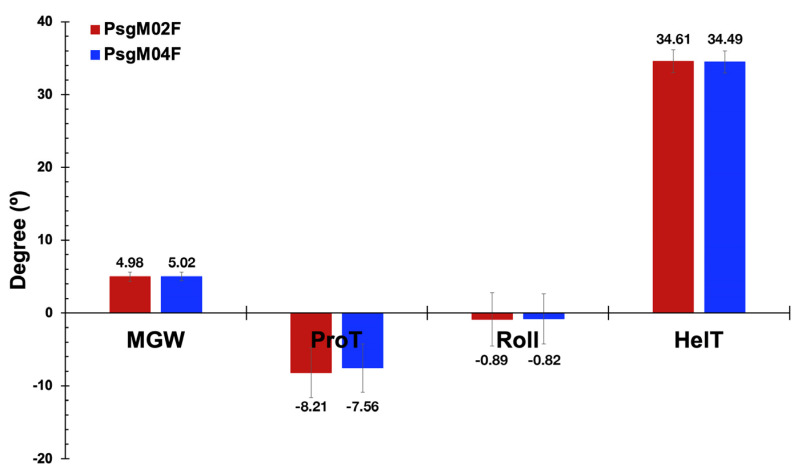
Statistical characteristics of the four predicted structural features from the PsgM02F and PsgM04F phage genomes.

In a previous work, the two lytic polyvalent phages displayed very different characteristics in the adsorption, infection, and virion morphogenesis processes [12] within their isolation host strain (Psg IBSBF-158). Hence, studying the mechanical properties of their genomes (both myoviruses) was essential for a better and deeper understanding of the enormous variability in the infection rates of their target bacterial cells with concomitant very discrepant yields of the virion morphogenesis process within infected cells (viz. 124 virions per host cell (phage PsgM02F) vs. 12 virions per host cell (phage PsgM04F), results from Silva et al. [12]). Figure 5 displays the statistical characteristics of the four predicted structural features from the PsgM02F and PsgM04F phage genomes.

The results displayed in Figure 5, pertaining to the four predicted structural features of the two-phage genomes, allow to observe the average values of MGW (max: 6.20; min: 2.85); phage PsgM02F: 4.98 ± 0.63; PsgM04F: 5.02 ± 0.57), ProT (max: −0.03; min: −16.51); phage PsgM02F: −8.21 ± 3.42; PsgM04F: −7.56 ± 3.31), Roll (max: 8.64; min: −8.57); phage PsgM02F: −0.89 ± 3.66; PsgM04F: −0.82 ± 3.43) and HelT (max: 38.05; min: 30.98); phage PsgM02F: 34.61 ± 1.57; PsgM04F: 34.49 ± 1.53). Higher predicted ProT angle values in the genome of phage PsgM04F can be observed compared with those of phage PsgM02F (Figure 5). ProT is a metric for the variability of the angle between the planes of two nucleotide bases and is associated with the rigidity of the DNA helix, influenced by potential interbase-pair hydrogen bonds in the major groove. Hence, a more positive ProT angle indicates a more rigid helix; therefore, these results can suggest the presence of more rigid regions along the PsgM04F phage genome than in the PsgM02F phage genome.

MGW is vital to DNA–protein interactions, characterized by A and T residues and is typically associated with flexibility. The narrowness of the minor groove suggests a greater accessibility of nucleotide base edges to proteins such as transcription factors, as will be discussed later, which can form bond connections, leading to changes in the geometry of the dsDNA molecule and significantly impacting DNA flexibility. The PsgM02F phage genome displays an average MGW angle value of 4.98, while the PsgM04F phage genome has an average value of 5.01 (Figure 5); hence, the PsgM02F phage genome may exhibit more flexible regions along its dsDNA molecule due to the narrower MGW than the PsgM04 phage genome.

The average predicted Roll angle value is −0.88 for the PsgM02F phage genome and −0.81 for the PsgM04F phage genome (Figure 5), but the genome of phage PsgM02F displays more positive Roll values than the genome of phage PsgM04F. Roll is associated with dsDNA bending into the grooves. Hence, one can deduce that the PsgM02F phage genome demonstrates greater flexibility in comparison to the PsgM04F phage genome.

**Figure 6:**
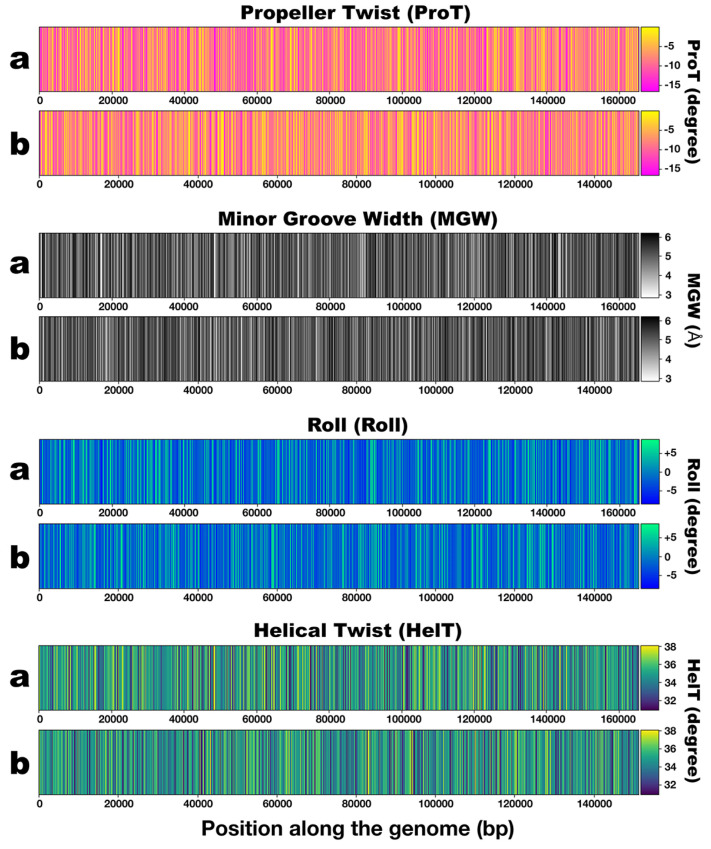
Heatmap patterns from the results of DNA shape (Propeller Twist, Minor Groove Width, Roll, and Helical Twist) calculations for the assembled genomes of phage PsgM02F (**a**) and phage PsgM04F (**b**).

Figure 6 displays the results from DNA shape (Propeller Twist, Minor Groove Width, Roll, and Helical Twist) calculations for the assembled genomes of the two phages.

The ProT heatmap of phage PsgM02F (Figure 6a: ProT) displays a lot more magenta patterns than that of phage PsgM04F (Figure 6b: ProT), denoting lower ProT angle values in the phage PsgM02F genome than in the genome of phage PsgM04F.

The MGW genomic heatmaps exhibit black and white patterns distributions, indicating higher and lower MGW predicted angles, respectively (Figure 6a,b: MGW). The MGW heatmap of phage PsgM02F genome displays less blackish patterns than that of phage PsgM04F, implying higher MGW angle values in the genome of phage PsgM04F than in the genome of phage PsgM02F.

The Roll heatmaps for both PsgM02F and PsgM04F phage genomes display a characteristic pattern distribution. The genome of phage PsgM02F displays more greenish patterns (hence more positive Roll values) than the genome of phage PsgM04F (which displays more blueish patterns, hence more negative Roll values) (Figure 6a,b: Roll).

**Figure 7:**
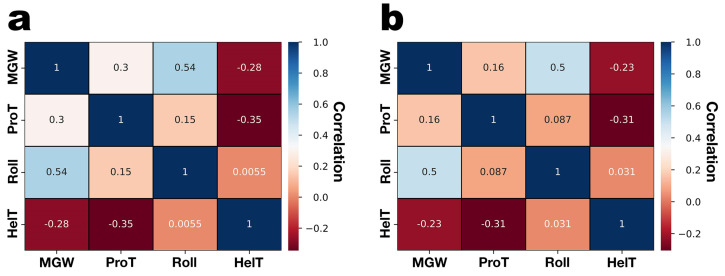
Correlation between the four predicted structural features of the genomes of phage PsgM02F (**a**) and phage PsgM04F (**b**).

Figure 7 displays the correlations between the four predicted structural features of the PsgM02F and PsgM04F phage genomes.

Positive and negative correlations can be observed in both phage genomes (Figure 7a,b). Interestingly, the correlation among DNA shape features appears comparable between genomes. A higher correlation between Roll and ProT can be observed for the genome of phage PsgM02F (Figure 7a) compared to that of phage PsgM04F (Figure 7b). In both phage genomes, a positive correlation is shown between the MGW and the Roll angle.

On the other hand, a negative correlation is observed between the MGW and the helix twist (HelT) for both phage genomes. A negative correlation between ProT and HelT is also shown in both phage genomes (Figure 7a,b).

**Figure 8:**
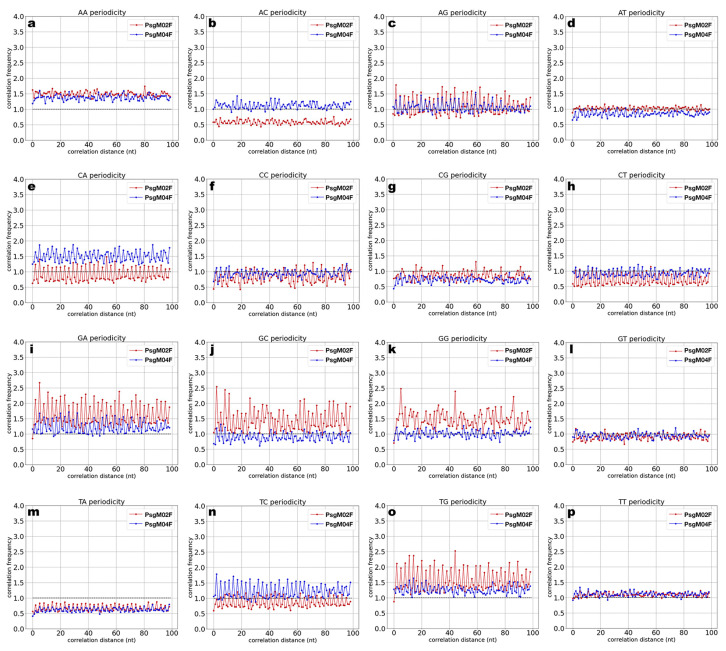
Dinucleotide correlation frequency patterns in the assembled genomes of phages PsgM02F and PsgM04F. (**a**) AA dinucleotide, (**b**) AC dinucleotide, (**c**) AG dinucleotide, (**d**) AT dinucleotide, (**e**) CA dinucleotide, (**f**) CC dinucleotide, (**g**) CG dinucleotide, (**h**) CT dinucleotide, (**i**) GA dinucleotide, (**j**) GC dinucleotide, (**k**) GG dinucleotide, (**l**) GT dinucleotide, (**m**) TA dinucleotide, (**n**) TC dinucleotide, (**o**) TG dinucleotide, (**p**) TT dinucleotide. Red line: phage PsgM02F; Blue line: phage PsgM04F.

Figure 8 displays the results obtained in the dinucleotide correlation frequency calculations performed on the two-phage genomes. With these calculations, one aimed to check the presence of peak patterns, meaning periodicity. The *x*-axis in the plots in Figure 8 represents the number of incremental steps where one can observe such periodicity.

A close inspection of the plots in Figure 8 allows us to observe that AA dinucleotide exhibits a higher presence in both phage genomes in 100 steps when compared to the random expected sequence (dotted line) without any discernible periodicity (Figure 8a). The AC dinucleotide (Figure 8b) in the phage PsgM02F genome has a frequency that suggests a lesser occurrence than expected. On the other hand, the AC dinucleotide in phage PsgM04F genome displays a frequency similar to the random expected sequence, but neither genome shows periodicity for this dinucleotide. No periodicity is observed for the AG dinucleotide in both phage genomes (Figure 8c). In the genome of phage PsgM02F, AT frequency is aligned with the random expected sequence (Figure 8d), while in the genome of phage PsgM04F, the frequency of AT indicates a smaller occurrence than expected by random.

In the genome of phage PsgM02F, CA frequency is marked by peaks fluctuating around the random expected sequence; however, it does not display periodicity. On the other hand, in the genome of phage PsgM04F, the frequency of CA is elevated when compared to the random sequence but without displaying periodicity. Both CC (Figure 8f) and CG (Figure 8g) in the genome of phage PsgM02F show no discernible periodicity. On the other hand, CG in the genome of phage PsgM04 (Figure 8g) has a diminished presence compared to the random sequence.

GA, GC, and GG dinucleotides in the genome of phage PsgM02F display the highest frequencies (Figure 8i–k).

TA in both phage genomes (Figure 8m) is sparsely represented, whereas TC shows an elevated presence in the genome of phage PsgM04F (Figure 8n).

In both phage genomes, TG is above the random expected sequence, indicating a higher presence. Lastly, GT (Figure 8l) and TT (Figure 8p) exhibit neither representation nor periodicity in both phage genomes.

The over- and under-representation of these dinucleotides in both phage genomes is variable. Remarkably, AA is over-represented in both phage genomes, with a more pronounced correlation frequency in the genome of phage PsgM02F (Figure 8a) when compared to the genome of phage PsgM04F.

On the other hand, GC and GG show a substantial over-representation in the genome of phage PsgM02F (Figure 8j,k) but not in the genome of phage PsgM04F. The distribution of these dinucleotides across 100 steps implies a higher degree of bendability in the PsgM02F phage genome when compared to the PsgM04F phage genome.

Taking together, the analysis of the dinucleotide distance correlation in both phage genomes reveals variabilities in the occurrence of specific dinucleotides within 100 steps in both phage genomes. However, no striking oscillations or discernible periodic patterns are noticeable for these particular dinucleotides in either genome. GG in the genome of phage PsgM02F displays potential periodicity every 38 steps, suggested by the presence of three distinctive peaks at that interval. However, further studies are required to validate this observation.

**Figure 9:**
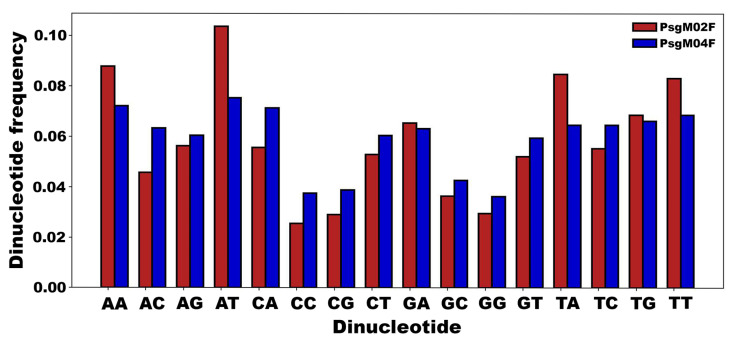
Dinucleotide frequency in the genomes of phages PsgM02F and PsgM04F.

A Python script was used to investigate the occurrence of the possible 16 dinucleotides (AA, AC, AG, AT, CA, CC, CG, CT, GA, GC, GG, GT, TA, TC, TG, and TT) in the genomes of phages PsgM02F and PsgM04F. This was accomplished by counting the occurrences of each dinucleotide in each phage genome and dividing them by the respective genome’s length. Figure 9 displays the Dinucleotide frequency in the genomes of phages PsgM02F and PsgM04F.

Variations were unveiled in the occurrence of distinct dinucleotides in the two-phage genomes (Figure 9). In the genome of phage PsgM02F, dinucleotides AA, AT, TA, and TT exhibited the highest frequencies when compared with the genome of phage PsgM04F (Figure 9).

**Figure 10:**
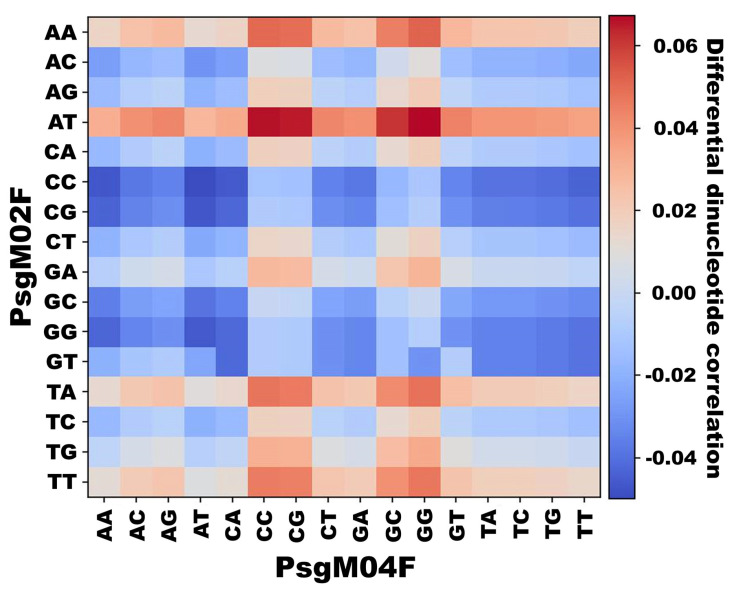
Heatmap of the differential dinucleotide frequencies between the genomes of phages PsgM02F and PsgM04F.

Initially, one independently determined the frequency of all 16 possible dinucleotide combinations in both phage PsgM02F and PsgM04F genomes (Figure 9). Subsequently, the difference in dinucleotide frequencies between phage PsgM02F and PsgM04F genomes was computed by counting the occurrence of each dinucleotide in each phage genome and dividing it by the respective genome length, followed by determining the difference in dinucleotide frequencies between the genomes of phages PsgM02F and PsgM04F and representing it as a heatmap (Figure 10) where a high differential frequency can be seen depicted in red, whereas a small differential frequency can be seen depicted in blue. Figure 10 displays the heatmap of the differential dinucleotide frequencies between the genomes of phages PsgM02F and PsgM04F.

Figure 10 reveals a significant contrast in the prevalence of AA, AT, TA, and TT in the genome of phage PsgM02F compared to every other dinucleotide in the genome of phage PsgM04F. Overall, these specific dinucleotides are quite more abundant in the genome of phage PsgM02F than any dinucleotide in the genome of phage PsgM04F.

**Figure 11:**
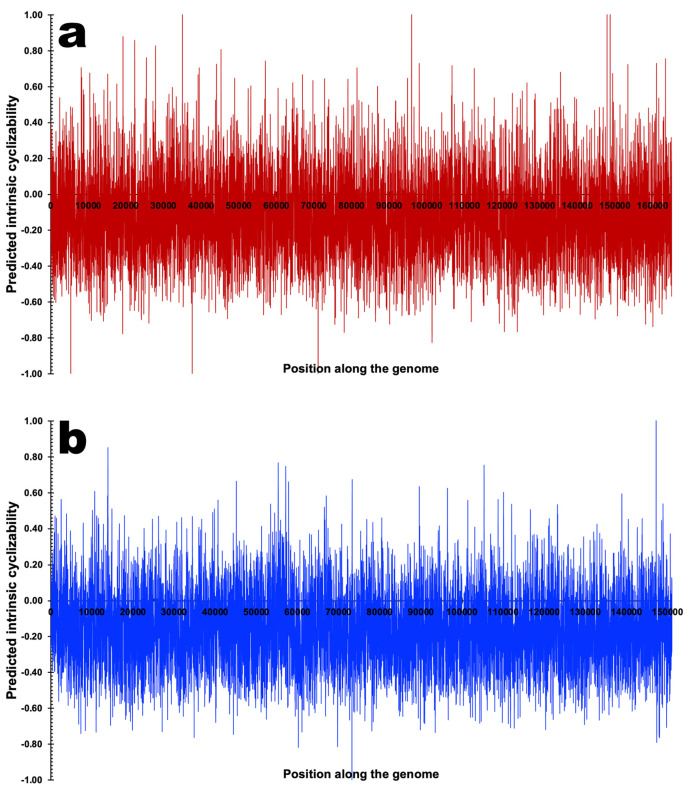
Predicted intrinsic cyclizability along the phage genome at 7 bp resolution for (**a**) phage PsgM02F and (**b**) phage PsgM04F.

In Figure 11, the predicted intrinsic cyclizability along the phage genome at 7 bp resolution can be observed for phage PsgM02F and phage PsgM04F.

Both phage genomes exhibit varying cyclizability, represented as high and low peaks in the plots of Figure 11.

**Figure 12:**
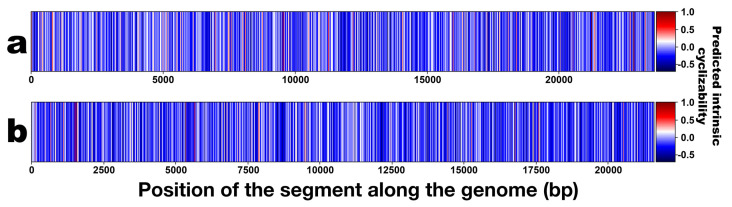
Heatmap patterns of the cyclizability values of the genomes of phages PsgM02F (**a**) and PsgM04F (**b**).

Figure 12 displays the heatmap patterns of the predicted intrinsic cyclizability values of the genomes of phages PsgM02F and PsgM04F.

The degree of flexibility in the two-phage genomes, implied by the number of peaks in Figure 11a,b, is visually supported by their corresponding heatmaps (Figure 12a,b), where red denotes high cyclizability and blue denotes low cyclizability. The two-phage genomes demonstrate a different degree of intrinsic cyclizability (Figure 12).

**Figure 13:**
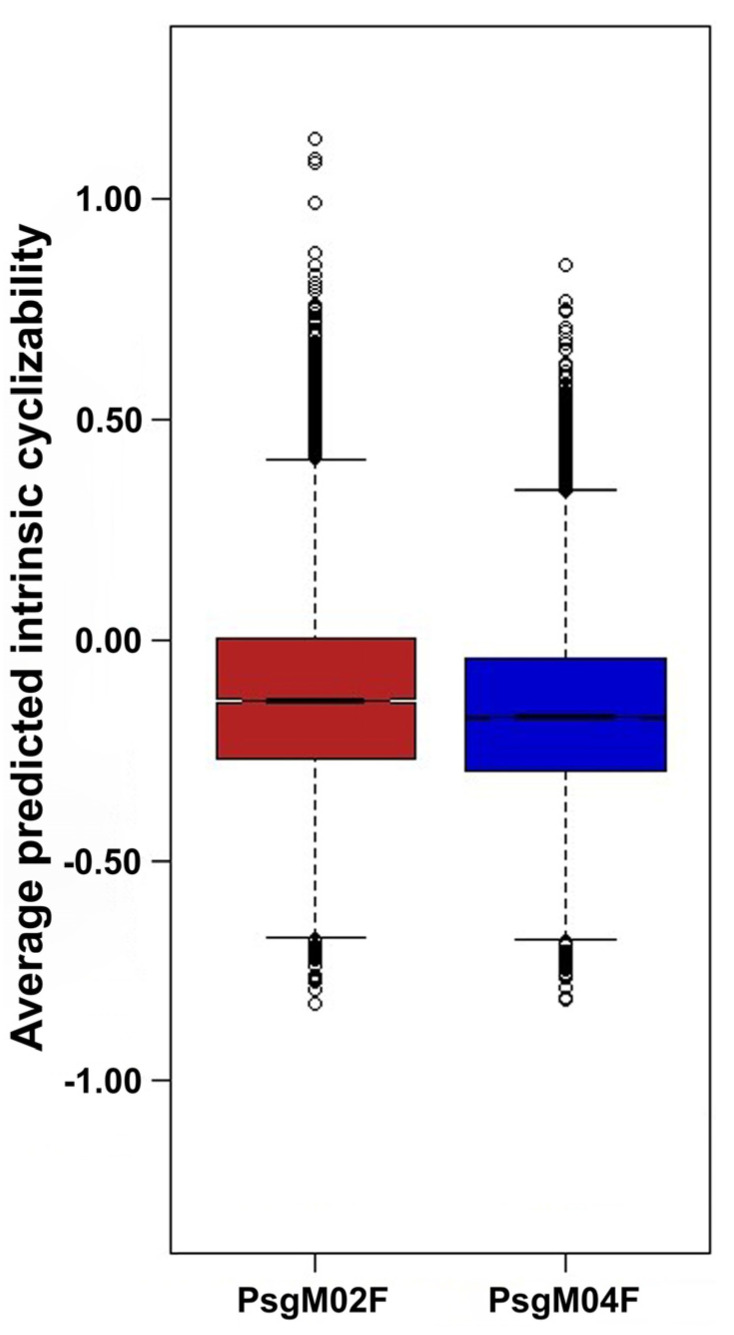
Distribution of predicted intrinsic cyclizabilities of phage PsgM02F and phage PsgM04F genomes, showing its statistics.

Figure 13 allows us to observe box plots with the statistics of the cyclizabilities of both phage genomes.

The mean range of intrinsic cyclizability is indicated by the black line within each box plot in Figure 13, where the maximum and minimum whiskers indicate the highest and lowest cyclizability values. Outliers are shown in open circles.

A close inspection of the data in Figure 13 allows relatively large standard deviations of the mean predicted cyclizabilities to be observed, and the average predicted intrinsic cyclizabilities of phages PsgM02F and PsgM04F genomes were checked and found to be statistically different between them. A one-way ANOVA statistical analysis was performed on the whole set of genome-predicted intrinsic cyclizability data to test the null hypothesis that the average predicted intrinsic cyclizabilities of the two-phage genomes were similar. The results obtained are displayed in Table 3.

The F-ratio allows for the question to be answered as to whether the variance between the means of the predicted intrinsic cyclizability populations of the two genomes were significantly different, whereas the *p*-value is the probability of getting average predicted intrinsic cyclizabilities at least as extreme as the ones that were actually observed in the genomes of the two phages.

The web-based ViPTree program (https://www.genome.jp/viptree (accessed on 6 January 2024)) [78] was used to analyze similarities and relationships between phages PsgM02F and PsgM04F and other prokaryotic dsDNA viruses, as described by Xuan and colleagues [79]. All computations were performed using the SuperComputer System of the Institute for Chemical Research of Kyoto University (Kyoto, Japan).

**Figure 14:**
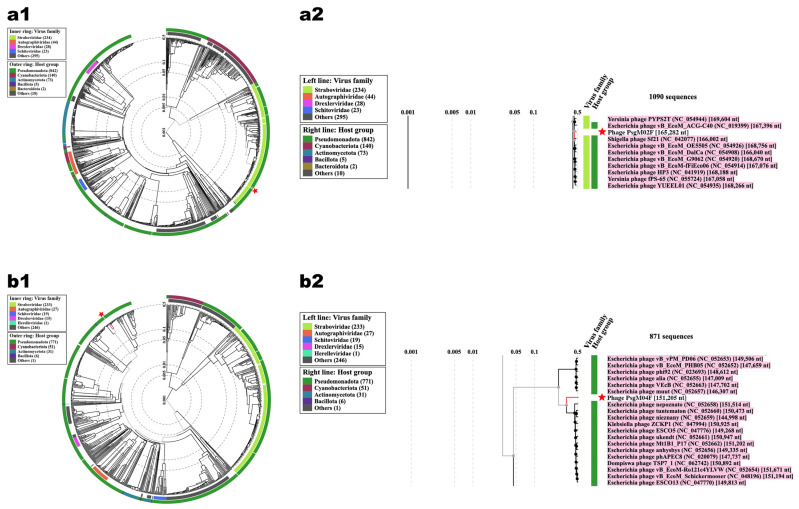
Viral proteomic trees resulting from ViPTree analyses of phages PsgM02F (**a1**,**a2**) and PsgM04F (**b1**,**b2**) and related phages. (**a1**,**b1**) A total of 5633 phage genomes were used as reference sequences to build phylogenetic trees using ViPTree. This figure identifies phages according to their official ICTV classification, with the inner and outer rings indicating their virus family and host group, respectively. (**a2**,**b2**) Expanded views of the regions of the trees containing the most closely related phages. The branches containing phage PsgM02F (**a2**) and phage PsgM04F (**b2**) are displayed in red. Red star pinpoints the location of phages PsgM02F (**a1**,**a2**) and PsgM04F (**b1**,**b2**).

Figure 14 displays the viral proteomic trees resulting from ViPTree analyses of phages PsgM02F (Figure 14(a1,a2)) and PsgM04F (Figure 14(b1,b2)) and related phages.

Results showed that phage PsgM02F was grouped into one small group with 10 other phages (Figure 14(a2)), with nearly all such phages belonging to the *Straboviridae* family according to the updated ICTV taxonomic classification, whereas phage PsgM04F was grouped into one small group with other 19 phages (Figure 14(b2)).

## 4. Discussion

The development of new (feasible) environmentally friendly antibacterial alternatives to conventional copper- and antibiotic-based treatments aiming at controlling infections by *Pseudomonas syringae* pv. *garcae* (Psg) in coffee plantations has been quite challenging. The present study has two previously isolated new polyvalent lytic phages for *Pseudomonas syringae* pv. *garcae* (viz. PsgM02F and PsgM04F) were further characterized relative to their virion morphogenesis yield and had their genomes fully characterized from both molecular and mechanical points of view.

Phages PsgM02F and PsgM04F form dimensionally different and clear plaques on the host lawn, with diameters ranging from ≈0.5 to ≈1.5 mm, respectively (Figure 1a,d). Further characterization by transmission electron microscopy and whole genome sequencing confirmed both phages as myoviruses (phage morphotypes with a contractile tail) with different genus members (Figure 1b,c,e,f; Table 2). Both phages belong to the class Caudoviricetes (phage PsgM02F: Figure 1b,c; phage PsgM04F: Figure 1e,f) and, while phage PsgM02F belongs to subfamily *Straboviridae*, genus *Tequatrovirus*, phage PsgM04F belongs to subfamily *Stephanstirmvirinae*, genus *Phapecoctavirus* (Table 2), according to recent bacterial viruses’ taxonomy [77]. The taxonomic lineage of phage PsgM02F was therefore established as *Viruses* > *Duplodnaviria* > *Heunggongvirae* > *Uroviricota* > *Caudoviricetes* > *Straboviridae* > *Tevenvirinae* > *Tequatrovirus*, whereas the taxonomic lineage of phage PsgM04F was established as *Viruses* > *Duplodnaviria* > *Heunggongvirae* > *Uroviricota* > *Caudoviricetes* > *Stephanstirmvirinae* > *Phapecoctavirus* [80,81]. The coding sequences (CDS) with predicted functions among the 278 annotated CDS in the genome of phage PsgM02F, or among the 324 annotated CDS in the genome of phage PsgM04F (Table 2), did not encode depolymerase enzymes, toxins, virulence factors, antibiotic resistance, or integrase enzymes (Supplementary Table S1), with typical structural proteins such as capsid, tail, baseplate and spike proteins together with DNA metabolism-related proteins being annotated in the two-phage genomes (Figure 2). Since no lysogenic-related (integrase) genes were found in the genomes of both phages, following whole phage genome sequencing and annotation [82], one can safely assume that phages PsgM02F and PsgM04F follow a lytic lifestyle [35,36,83] and are, therefore, adequate for Psg control trials.

While myoviruses of the genus *Tequatrovirus*, family *Straboviridae*, are characterized by several unique features, viz. contractile tails and linear double-stranded DNA (dsDNA) encased within a relatively large capsid and a long, thick, complex, contractile tail consisting of a central tube (built of stacked rings of six subunits and surrounded by a helical contractile sheath that is separated from the capsid by a neck) and ancillary structures (tail fibers), myoviruses of the genus *Phapecoctavirus*, family *Stephanstirmvirinae*, are characterized by isometric capsids with a roughly spherical shape, a long, non-flexible tail with a contractile sheath surrounding a central tube and a linear dsDNA genome up to 160–175 kbp, making them larger than many other known bacteriophages [84,85]. These structural and genomic features are well observed in the TEM photomicrographs in Figure 1b,c,e,f together with their respective dimensions (Table 1 and Table 2).

Results from previous work [12] indicated that, besides their isolation host strain, both phages were able to bind to a few other bacterial species probably because they displayed some surface receptors that were recognized by these phages and killed them, although the EOP (%) values produced were in general quite small (phage PsgM02F: *Escherichia coli* ATCC 25922 (0.1110%), *Pseudomonas syringae* pv. *actinidiae* CRA-FRU 14.10 (0.00014%), *Proteus penneri* (0.00041%), *Proteus vulgaris* CCCD-P002 (0.00008%); phage PsgM04F: *Escherichia coli* ATCC 25922 (0.0205%), *Enterococcus faecalis* CCCD-E002 (0.0036%), *Pseudomonas syringae* pv. *actinidiae* CRA-FRU 14.10 (440%). On one bacterial species, however, phage PsgM04F infection was highly productive, resulting in a large number of progeny virions and yielding a large EOP (440%). This was the case for *Pseudomonas syringae* pv. *actinidiae* CRA-FRU 14.10 [12]. According to Hyman [82], (newly) isolated phage particles may be able to infect host cells from different species displaying the same general type(s) of receptors on their surface as the isolation host. Knowing that there are a few “true” polyvalent phages that infect across bacterial genera, using a bacterial pilus protein as their receptor so they infect many species that happen to have the plasmid for a particular pilus, the two phages characterized in the present research effort might be indeed polyvalent [86,87,88,89,90,91,92].

According to a mechanistic rationale deployed by Balcão et al. [30], dsDNA sequences rich in GC dinucleotides are less flexible (i.e., more rigid) than sequences rich in AT dinucleotides. Despite having a higher net 42.3% GC (and 57.7% AT) content in its genome (Table 2), phage PsgM04F dsDNA sequences are probably rich(er) in GC dinucleotides (thus imparting a more rigid genome) than the genome of phage PsgM02F, which is in clear agreement with the (much lower) value obtained for the virion morphogenesis yield of phage PsgM04F [12]. Hence, the genome of phage PsgM02F is apparently more flexible than the genome of phage PsgM04F, which is in clear agreement with the values obtained for the virion morphogenesis yield of both phages with phage PsgM04F (integrating a more rigid genome) producing a much smaller virion morphogenesis yield than phage PsgM02F. These observations are consistent with the rationale that upon translocation of a less bendable dsDNA genome into the bacterial host cytoplasm, the bacterial host RNA polymerase fails to interact and negotiate with it in an efficient way, leading to lower transcription rates and production of phage proteins in smaller numbers with concomitant assembly of a small number of mature virions [30].

*DNA Structural features*. The structural integrity of the DNA molecule relies on the nucleotides, exhibiting various degrees of freedom, including bending, twisting, and compression. It is widely acknowledged that the shape of the DNA molecule (consisting of physical and geometrical properties such as the width of the minor groove (MGW), Helical Twist (HelT), Propeller Twist (ProT) and Roll) significantly influences its specific interaction with various proteins. These characteristics can be predicted based on the DNA base pair sequence, unveiling inherent preferred conformations [93,94]. Meanwhile, Propeller Twist (ProT) involves the rotation of one nucleotide base with respect to the other in the same base pair. On the other hand, Helical Twist (HelT) describes rotation with respect to the helical axis.

In this work, DNA shape was predicted for both phage genomes (PsgM02F and PsgM04F) and visually represented as heatmaps (Figure 6). The *x*-axis denotes the number of nucleotides per genome sequence, while the *y*-axis illustrates the predicted angle values per property.

Throughout the genome sequences, higher predicted angle values in the ProT heatmap of phage PsgM04F can be observed compared with those in the ProT heatmap of phage PsgM02F (Figure 5 and Figure 6). The higher values are represented as yellow sections in the corresponding heatmaps (Figure 6a,b, “Propeller Twist”). An average ProT angle value of −8.21 is displayed in the PsgM02F genome, contrasting with a −7.57 angle value for the PsgM04F genome (Figure 5 and Figure 6a,b, “Propeller Twist”). As can be clearly observed in the ProT heatmap plots of both phage genomes (Figure 6), the heatmap of phage PsgM02F has a lot more magenta patterns (ProT angles lower than −15°) than the one from phage PsgM04F, implying that the ProT angles in the genome of phage PsgM02F are much more negative than those in the genome of phage PsgM04F and, therefore, allows to conclude that the genome of phage PsgM02F is somehow more flexible than that of phage PsgM04F. A non-parametric Mann–Whitney U-test for non-normally distributed data was performed to assess the statistical difference between the ProT-predicted values obtained from both phage genomes. The results indicated a significant difference between the ProT values of the two-phage genomes, with a *p*-value < 0.05.

ProT serves as a metric for the variability of the angle between the planes of two nucleotide bases and is associated with the rigidity of the DNA helix, influenced by potential interbase-pair hydrogen bonds in the major groove [93]. Consequently, a more positive ProT angle indicates a more rigid helix [94]. Building on this observation and based on the previous results (Figure 5), it can be suggested that, on average, there are more rigid regions along the PsgM04F phage genome than the PsgM02F phage genome.

The MGW is commonly linked and vital to DNA–protein interactions [95]. This groove, characterized by the presence of A and T residues, is typically associated with flexibility [96]. A narrow minor groove enhances negative electrostatic potential, creating favorable conditions for interactions with positively charged amino acids from proteins. The narrowness of the minor groove suggests greater accessibility of nucleotide base edges to proteins such as transcription factors [93]. Transcription factors and various other proteins can form bond connections, altering the geometric characteristics of the dsDNA molecule [97,98]. This process can significantly impact DNA flexibility.

The genomic heatmaps exhibit distributions of black and white patterns, indicating higher and lower MGW predicted angles, respectively (Figure 6a,b, “Minor Groove Width”). The PsgM02F phage genome displays an average MGW angle value of 4.98, while the PsgM04F phage genome has an average value of 5.01 (Figure 5). Statistical analysis using the Mann–Whitney U-test showed us that the MGW in both phage genomes is statistically different, with a *p*-value = 1.12 × 10^−12^. Therefore, it can be inferred that the PsgM02F phage genome may exhibit more flexible regions along its dsDNA molecule due to the narrower MGW than the PsgM04 phage genome.

The heatmaps of the Roll structural feature for both PsgM02F and PsgM04F phage genomes display a characteristic pattern distribution. The color scheme uses green to denote higher angle values and dark blue to indicate lower values (Figure 6a,b, “Roll”). Specifically, the average predicted Roll angle value is −0.88 for the PsgM02F phage genome and −0.81 for the PsgM04F phage genome (Figure 5), but the genome of phage PsgM02F displays more greenish patterns (hence more positive Roll values) than the genome of phage PsgM04F (Figure 6a,b, “Roll”). A Mann–Whitney U-test revealed that the distribution of Roll angle values along both phage genomes is statistically different, with a *p*-value = 1.78 × 10^−48^.

Roll is associated with dsDNA bending into the grooves. Notably, significant positive Roll values in base pair steps imply weak stacking interactions, suggesting a higher degree of conformational flexibility [93]. The average values in our dataset do not indicate a positive angle. Nevertheless, the magnitude of the average value for the PsgM02F phage genome is higher and statistically different than that of the PsgM04F phage genome. Consequently, we can deduce that the PsgM02F phage genome demonstrates greater flexibility in comparison to the PsgM04F phage genome. This stands in support of the observations made for the ProT values and Roll values.

Upon completion of DNA structural feature calculations and heatmap generation, a pairwise correlation analysis was conducted to find Pearson’s correlation coefficient. This analysis aimed to unveil the linear relationships among the structural values (Figure 7a,b). In both correlation matrices (a: phage PsgM02F genome; b: phage PsgM04F genome), each variable is depicted by a row and a column, with the cells displaying the correlation between them. Positive and negative correlations can be observed in both phage genomes. Interestingly, the correlation among DNA shape features appears comparable between genomes. A higher correlation between Roll and ProT can be observed for the genome of phage PsgM02F (0.15, Figure 7a) compared to that of phage PsgM04F (0.087, Figure 7b).

In both phage genomes, a positive correlation is shown between the MGW and the Roll angle, with a correlation coefficient of 0.54 in the PsgM02F phage genome and 0.50 in the PsgM04F phage genome. This correlation can be explained because Roll angles measure the extent to which the best mean planes through two successive base pairs open towards the minor groove. Therefore, more positive Roll angles could indicate more positive MGW angles [99].

On the other hand, a negative correlation is observed between the MGW and the helix twist (HelT), with a value of −0.28 for the PsgM02F phage genome and −0.23 for the PsgM04F phage genome. According to a study by Liebl et al. [100], the overwinding of DNA decreases the size of the MGW. The resulting base pair inclination leads to an extended HelT due to the increase in the projection of the stacking distance between neighboring base pairs; therefore, we can infer a negative correlation between those features. This might explain our observations.

A negative correlation between ProT and HelT is also shown in both phage genomes (Figure 7, with a value of −0.35 for the PsgM02F phage genome and −0.31 for the PsgM04F phage genome). The purpose of propeller twisting is to optimize stacking interactions, and our results indicate a certain degree of correlation with the HelT. When values approach 36 degrees, ProTs are close to zero, meaning satisfactory stacking interactions. Deviations from this ideal result in reduced stacking, compensated for by ProTs. Additionally, El Hassan and Calladine [101] reported that more negative dsDNA ProT angles are associated with highly flexible dsDNA. In contrast, ProT angles ranging from −1 degrees to −3 degrees are linked to more rigid dsDNA.

Our findings align with the study conducted by Yella et al. [102], wherein they evaluated the correlation between DNA structural features using Pearson’s coefficient to determine the flexibility of flanking DNA.

Based on these observations, we can conclude that these different structural features interact to a certain degree, influencing the mechanical properties of dsDNA, such as DNA flexibility and rigidity. Our results suggest that the PsgM02F phage genome may have more flexible regions than the PsgM04F phage genome. These flexible regions are likely influenced by the different structural features discussed in this section: ProT, MGW, HelT, and Roll.

Such structural features could potentially play a role in the infection of, and translocation of phage dsDNA into, susceptible bacterial host cells. A narrow MGW, a distinct HelT conformation, alterations in Roll, and variations in ProT may collectively impact the entire process by facilitating enhanced DNA–protein interactions, improving accessibility, and contributing to the stability of the DNA structure during translocation events. The results allow us to partially explain the differences observed in the phage virion morphogenesis yields within infected Psg IBSBF-158 host cells. While our findings imply a potential advantage for the PsgM02F phage genome over the PsgM04F phage genome, in-depth analysis is required to substantiate these observations. While the DNA molecule is a relatively rigid biopolymer, mechanical deformations such as bending are ubiquitous [34,102] in the molecule. DNA ProT angles lower than −30° are usually related to very flexible DNA, whereas ProT angles between −1° and −3° are usually related to less flexible DNA [34].

*Dinucleotide distance correlation of both PsgM02F and PsgM04F phage genomes*. A dinucleotide refers to the pairing of two nucleotides along the DNA sequence. The observed transitions of dinucleotides can be correlated with the thermodynamic, geometrical, and structural properties of DNA sequences [103].

The occurrence frequency of a dinucleotide within a sequence is commonly used as a genomic signature for various microorganisms [104]. The product of the corresponding nucleotide occurrences determines the anticipated frequency of finding a dinucleotide. The dinucleotide frequency is the incidence of a given neighbor dinucleotide in a DNA sequence. When all nucleotides are assessed randomly, the frequency of each of the 16 possible dinucleotide pairs should be the same [105]. In an unbiased sequence, the repetition of observed dinucleotides aligns with the expected repetition, resulting in a ratio (observed/expected) equal to 1 [106]. Consequently, a ratio higher than 1 indicates overexpression of the specific dinucleotide, while a ratio less than 1 signifies under-expression throughout the sequence [107].

If a dinucleotide occurs at a consistent, repetitive distance within a sequence, we can say that periodicity is observed. Wu et al. [108] have documented that specific dinucleotides exhibit periodicity, correlated with DNA flexibility. According to their research, periodicity is identified approximately every 10 base pairs, aligning with the pitch of the DNA helix, which ranges from 9.7 to 11 base pairs, depending on the sequence. However, periodicity, in general, can be identified at any repetitive distance within a sequence.

It is well known that dsDNA flexibility depends on its sequence, which affects dsDNA stability under bending. Sequence-dependent flexibility is an important characteristic that guides DNA-binding proteins to targets and is vital for DNA looping and transcription factor binding [109], with some studies suggesting that there is a correlation between flexibility and dinucleotide frequency [109]. According to Basu et al. [74], the spatial arrangement of certain dinucleotides might play a crucial role in determining the intrinsic cyclizability and flexibility of DNA. Utilizing high-throughput data, Basu and colleagues [74] have calculated the pairwise distance distribution function to quantitatively assess how specific dinucleotide pairs impact the mechanics of DNA, such as flexibility.

In this study, the self-pairwise distance distribution function was computed for the 16 possible dinucleotide combinations, viz. AA, AC, AG, AT, CA, CC, CG, CT, GA, GC, GG, GT, TA, TC, TG, and TT, in both phage genomes (Figure 8a–p), aiming at assessing dinucleotide periodicity, which is associated with DNA bendability [34]. A close inspection of each one of the plots in Figure 8 allows us to draw some conclusions. The AA dinucleotide exhibits a higher presence in both phage genomes in 100 steps compared to the random expected sequence (depicted by the dotted line) without displaying any discernible periodicity (Figure 8a). In the PsgM02F phage genome, the AC dinucleotide (Figure 8b) has a frequency of approximately 0.5 compared to the random expected sequence, suggesting a lesser occurrence than expected. Conversely, the AC dinucleotide in the PsgM04F phage genome exhibits a frequency similar to the random expected sequence, but neither genome shows periodicity for this dinucleotide. For the AG dinucleotide in both genomes (Figure 8c), no periodicity is observed, and the ratio fluctuates from 0 to 1, possibly attributed to noise. In the PsgM02F phage genome, the AT dinucleotide frequency aligns with the random expected sequence (Figure 8d), while in the PsgM04F phage genome, the AT dinucleotide frequency is almost 0.8, indicating a smaller occurrence than expected by random chance.

In the PsgM02F phage genome, the CA dinucleotide exhibits a frequency ranging from 0.7 to 1 (Figure 8e), marked by peaks fluctuating around the random expected sequence without displaying periodicity. Conversely, in the PsgM04F phage genome, the CA dinucleotide shows an elevated presence compared to the random sequence, with a frequency of almost 1.5, but does not exhibit periodicity. Both the CC (Figure 8f) and CG (Figure 8g) dinucleotides in the PsgM02F phage genome show no discernible periodicity and maintain a frequency of around 1, showing no particular representation in either genome. Conversely, the CG dinucleotide in the PsgM04 genome (Figure 8g) has a diminished presence in 100 steps compared to the random sequence.

In the PsgM02F phage genome, CT (Figure 8h) appears to have a minor occurrence compared to the random sequence, with a frequency value of approximately 0.8. However, the proximity of certain peaks to the random expected sequence may suggest the presence of noise. In contrast, in the PsgM04F phage genome, CT does not exhibit any distinct representation.

The GA, GC, and GG dinucleotides in the PsgM02F phage genome show the highest frequency, reaching around 1.8, with some peaks reaching almost 2.5 in frequency (Figure 8i–k). While this does not indicate periodicity, it does mean a significantly higher occurrence of these dinucleotides. There is a hint of vague periodicity in the GG dinucleotide in the PsgM02F phage genome (Figure 8k), as evidenced by the observation of three large peaks every 38 steps; however, further analysis is needed to confirm this pattern. In the PsgM04F phage genome, the GA dinucleotide surpasses the random expected sequence, unlike the GC and GG dinucleotides, which do not exhibit a discernible representation and appear as noise.

The TA dinucleotide in both PsgM02F and PsgM04F phage genomes (Figure 8m) is sparsely represented, with a frequency of around 0.7. The TC dinucleotide shows an elevated presence in the PsgM04F phage genome (Figure 8n), but the representation is not clear, like in the PsgM02F phage genome, where a minor representation is observed.

The TG dinucleotide in the PsgM02F phage genome (Figure 8o) has a frequency of around 1.8, while in the PsgM04F phage genome, the frequency is around 1.2. In both genomes, this dinucleotide is above the random expected sequence, indicating a higher presence. Lastly, GT (Figure 8l) and TT (Figure 8p) exhibit neither representation nor periodicity in both phage genomes.

The bias of these dinucleotides from the expected sequence, as indicated by the dotted line, reveals a notable variation in dinucleotide composition across both phage genomes. Basu et al. [74] have reported the significant role of dinucleotides in DNA flexibility. According to findings by Basu et al. [74], Mrázek [110] and Wu et al. [108], dinucleotides consisting exclusively of adenines (A’s) or thymines (T’s) positively contribute to intrinsic cyclizability and DNA flexibility. This holds true for dinucleotides containing guanines (G’s) or cytosines (C’s) as well, including AA, AT, TA, TT, CC, CG, GC, and GG [74].

The over- and under-representation of these dinucleotides in both phage genomes is variable. Notably, the AA dinucleotide is over-represented in both phage genomes, with a more pronounced correlation frequency in the PsgM02F phage genome (Figure 8a) compared to the PsgM04F phage genome. Conversely, the GC and GG dinucleotides show a substantial over-representation in the PsgM02F phage genome (Figure 8j,k) but not in the PsgM04F phage genome. The distribution of these dinucleotides across 100 steps implies a higher degree of flexibility in the PsgM02F phage genome compared to the PsgM04F phage genome.

According to Johnson et al. [111], the AT dinucleotide is recognized for its high flexibility, enhancing the bendability of DNA and exhibiting one of the highest tendencies for looping. In the PsgM02F phage genome, the AT dinucleotide is compared to the random expected sequence (Figure 8d), while in the PsgM04F phage genome, it is under-represented. AT-rich sequences (Figure 8d) in the PsgM02F phage genome have a lower persistence length and, therefore, are more flexible than GC-rich sequences (Figure 8j).

Conversely, TA emerges as a dinucleotide with a relatively lower occurrence at a specific distance in both phage genomes (Figure 8m). Packer et al. [112] have previously established that TA can impart special mechanical properties like DNA flexibility, and recent studies, such as that by Back et al. [113], further support this observation. Back et al. [113] found a correlation between the TA dinucleotide and DNA cyclizability, giving significance to its strong association with DNA flexibility.

The lower occurrence of TA compared to the random expected sequence in both phage genomes does not deny its contribution to DNA flexibility. Notably, in the PsgM02F phage genome, the occurrence of TA is higher than in the PsgM04F phage genome, implying a greater degree of flexibility for the former genome.

Lyubchenko et al. [114] conducted a study indicating that CA enhances DNA flexibility in certain regions. The PsgM04F phage genome exhibits a higher frequency of the dinucleotide CA in comparison to the PsgM02F phage genome, where it is less prevalent (Figure 8e). This observation leads to the hypothesis that the PsgM04F phage genome might possess greater flexibility in certain regions where CA is located than the PsgM02F phage genome. However, more studies are needed to fully ascertain this. CG exhibits a diminished representation or occurrence compared to the random expected sequence in both phage genomes (Figure 8g). Some studies propose that CG is susceptible to methylation/deamination mutations, leading to G.C → T.A mutation [115]. Another hypothesis suggests that CG suppression may be influenced by various aspects of DNA conformation, including secondary structures and dinucleotide stacking energies [115]. This phenomenon might be occurring in both phage genomes, where it is underrepresented; however, additional studies are necessary to substantiate this observation.

The analysis of the dinucleotide distance correlation in both phage genomes reveals variabilities in the occurrence of specific dinucleotides within 100 steps in both the PsgM02F and PsgM04F phage genomes. However, no striking oscillations or discernible periodic patterns are noticeable for these particular dinucleotides in either genome. It is worth mentioning that the GG dinucleotide in the PsgM02F phage genome displays potential periodicity every 38 steps, suggested by the presence of three distinctive peaks at that interval, although further studies are required to validate this observation.

Our analysis suggests that the occurrence of specific dinucleotides in the PsgM02F and PsgM04F phage genomes is intricately related to DNA flexibility to a certain degree. Numerous studies have established the impact of dinucleotides and their spatial arrangement within the genome on contributing to a more flexible DNA structure [103,108,116,117]. It is crucial to note, however, that the role of certain dinucleotides in DNA flexibility or rigidity is context-dependent, indicating that their influence is not universally defined and can vary.

Some studies, including those conducted by Langowski et al. [118], El Hassan and Calladine [117] and Wu et al. [108], have reported the connection between the studied dinucleotides and their interplay with one another, showing their role in influencing DNA flexibility. This insight is an aspect that we will incorporate into our considerations for future studies.

Dinucleotide frequency patterns in the viral genome of bacteriophages are linked to distinct functional elements within the phage viral genome [107]. Variations from the expected occurrence of dinucleotides could influence translocation into the cytoplasm of susceptible bacterial host cells. The advantages of a more flexible DNA over a rigid DNA may include potential effects on the functioning of the translocating machinery and the transcriptional machinery inside the host cell [30].

*Differential dinucleotide frequency between PsgM02F and PsgM04F phage genomes.* Given the results from the pairwise distance distribution function, we decided to investigate the frequency of occurrence of each one of the 16 dinucleotides along the phage genomes without considering the distance. For that, we used a custom Python script to calculate their occurrence. The resulting plot displays the frequencies of these dinucleotides on the *y*-axis, with separate bars representing phages PsgM02F and PsgM04F in red and blue, respectively (Figure 9). Then, we computed the differential frequency of each dinucleotide between the PsgM02F and PsgM04F phage genome (Figure 10). The *x*-axis represents the PsgM04F phage genome, and the *y*-axis represents the PsgM02F phage genome.

Our findings reveal variations in the occurrence of distinct dinucleotides. In the PsgM02F phage genome, dinucleotides AA, AT, TA, and TT exhibit the highest frequencies, measuring 0.088, 0.142, 0.085, and 0.083, respectively. The PsgM04F phage genome shows the lowest frequencies for dinucleotides AA, AT, and TA, with values of 0.072, 0.075, and 0.071, respectively (Figure 9).

A close inspection of Figure 10, depicting the differential frequency between the PsgM02F and PsgM04F phage genomes, reveals a significant contrast in the prevalence of AA, AT, TA, and TT compared to every other dinucleotide in the PsgM04F phage genome. Overall, these specific dinucleotides are much more abundant in the PsgM02F phage genome than any dinucleotide in the PsgM04F phage genome.

Basu et al. [74] have reported that dinucleotides comprising only A’s or T’s contribute positively to intrinsic cyclizability, as do dinucleotides containing G’s or C’s, such as AA, AT, TA, TT, CC, CG, GC, and GG. The PsgM02F phage genome exhibits a high representation of dinucleotides with A’s and T’s, while the PsgM04F phage genome has a higher representation of dinucleotides with G’s and C’s compared to PsgM02F. The presence of A in most of these dinucleotides could indicate a higher degree of flexibility due to the presence of the pyramidal ring and its conformational flexibility [119]; however, this is a speculative statement that needs to be proven with additional research. This information suggests a degree of contribution to cyclizability in both genomes, but the dinucleotides in the PsgM02F phage genome occur more frequently than those in the PsgM04F phage genome. Consequently, it can be inferred that those dinucleotides in the PsgM02F phage genome may contribute more to intrinsic cyclizability, which, in turn, might be more flexible than the PsgM04F phage genome.

This speculation aligns with previous studies reporting that TpA (meaning that T and A are on the same sequence, one next to the other, linked by a phosphate group; as opposite to TA, meaning that T and A are in different sequences) dinucleotide, known for its flexibility, is associated with significant DNA bending [120,121]. Additionally, short stretches of dA:dT dinucleotides (deoxyadenosine (A) pairs with deoxythymidine (DNA nucleoside T) in double-stranded DNA) cause bending toward the minor groove, further associating them with flexibility [122]. It is noteworthy that GC content is generally uncorrelated with intrinsic cyclizability unless it exceeds 65%. In our results, the GC dinucleotide has a low frequency of occurrence in both genomes (Figure 9), leading to the speculation that GC may not significantly contribute to intrinsic cyclizability in both phage genomes despite the fact that GC dinucleotides are normally more stable than AT [123]. In the study performed by Shishkin et al. [124], it was shown that GC has a high ring deformation energy, making the pair more rigid compared to AT [124].

*Cyclizability values of both PsgM02F and PsgM04F phage genomes.* Under physiological conditions, DNA undergoes continuous conformational changes, and among these changes, DNA bending can significantly influence genome regulation and packing [125]. A notable example is observed in the Lac operon in *Escherichia coli*, where looping plays a crucial role in the regulation of gene expression. Additionally, the intricate interactions between transcription factors and the DNA molecule can exemplify the impact of bending on genomic processes [126].

DNA flexibility is a crucial factor in the functioning of a myriad of cellular processes and can be assessed through both experimental and computational means via its cyclization tendencies. The efficiency of cyclization depends upon the length of the DNA molecule, with shorter base-pair fragments exhibiting higher flexibility. DNA cyclization was initially explored by Shore and Baldwin [127], who provided evidence of the helical nature of DNA, as reflected in the periodicity of cyclization efficiency [128]. Subsequently, various experimental and low-throughput methods have been developed to examine the cyclizability of DNA.

In 2021, Basu et al. [34] introduced a high-throughput method known as loop-seq to assess DNA cyclizability rate. The cyclizability of a sequence is characterized as the natural logarithm of the ratio of the likelihood of finding sequences in the looped group compared to a control group. The average value obtained from repeating loop-seq multiple times is called the intrinsic cyclizability, which measures DNA cyclization propensity or bendability. This mechanical property, intrinsic cyclizability, can be compared to the functional properties of the DNA molecule [27,34].

In the research work entertained herein, we have explored the intrinsic cyclizability of the PsgM02F and PsgM04F phage genomes at a resolution of 7 base-pair, following the procedures outlined by Basu et al. [74], where they simultaneously measured the intrinsic cyclizability of up to 90,000 different 50 base-pair DNA sequences.

Plots were generated depicting the predicted intrinsic cyclizability as a function of the position along the PsgM02F and PsgM04F phage genomes (Figure 11a,b). We can observe that both phage genomes exhibit varying cyclizability, represented as high and low peaks in the plots. The number of peaks in Figure 11a,b implies a degree of flexibility in the genomes, which is visually supported by the corresponding heatmaps (Figure 12a,b), where red denotes high cyclizability and blue denotes low cyclizability. The two-phage genomes demonstrate a different degree of intrinsic cyclizability, with an average of −0.12567 for the PsgM02F phage genome and −0.16482 for the PsgM04F phage genome (Figure 13). This observation was supported by a Mann–Whitney U-test, revealing a statistically significant difference in cyclizability between the PsgM02F and the PsgM04F phage genomes, with a *p*-value = 7.58 × 10^−88^, and also by a statistical ANOVA test (Table 3).

Overall, the net average predicted intrinsic cyclizability of phage PsgM04F genome is 1.312 times lower than that of phage PsgM02F genome, confirming that the genome of phage PsgM04F is, in fact, more rigid than the genome of phage PsgM02F. A close inspection of the one-way ANOVA results displayed in Table 3 allows us to observe that the *p*-value is less than α = 0.05 (or 5%) and *F*-ratio > *F*-critical. Therefore there is a significant difference between the average intrinsic cyclizabilities of the two-phage genomes. This means one can safely say that at least 95% of the time, a difference can be seen between the intrinsic cyclizabilities of the genomes of phages PsgM02F and PsgM04F. The critical value of *F* at a 95% probability level is much lower (3.842) than the observed value of *F* (428.585), which means that the null hypothesis is false. The intrinsic cyclizability data does suggest that the differences between the average intrinsic cyclizability seen within different groups (genomes of phages PsgM02F and psgM04F) are statistically significant.

Further examination of the maximum cyclizability values reinforces this distinction, with the PsgM02F phage genome reaching 1.1394 compared to 0.8523 for the PsgM04F phage genome. This finding implies that specific regions along the PsgM02F phage genome may consistently demonstrate higher cyclizability and bendability than those in the PsgM04F phage genome. These regions are likely associated with the unique DNA shape of each phage genome, as previously explored.

According to Basu et al. [34], sequences rich in TA dinucleotides tend to have high intrinsic cyclizability. This finding closely aligns with the observed higher frequency of TA in the PsgM02F phage genome of approximately 0.08 compared to that of the PsgM04F phage genome of approximately 0.06 (Figure 9). Consequently, regions with a higher occurrence of TA in both genomes may contribute to the observed cyclizability and, therefore, DNA flexibility. In addition, it has been previously established that the MGW is characterized by the presence of A and T residues, typically linked to flexibility [96]. The frequency of the dinucleotide AT and TA is higher in the PsgM02F phage genome than in the PsgM04F phage genome (Figure 9). Therefore, it is plausible to hypothesize that these dinucleotides contribute more flexibility in the PsgM02F phage genome than in the PsgM04F phage genome. This contribution may arise from the presence of AT and TA in the MGW, which, in turn, also contributes to its narrowness.

It is noteworthy that, despite the PsgM02F phage genome displaying higher cyclizability and bendability in contrast to the PsgM04F phage genome, the plots in Figure 11 reveal a greater number of negative peaks in the genome of phage PsgM02F compared to that of phage PsgM04F genome, having lower cyclizability values. This observation suggests the presence of rigid sections in specific areas along the PsgM02F phage genome. This statement may be connected to certain dinucleotides previously linked to rigidity, which exhibited higher occurrence in the PsgM02F phage genome when compared to the PsgM04F phage genome.

Basu et al. [74] reported a connection between intrinsic cyclizability and DNA shape in their study. Specifically, those researchers proposed that a high ProT is indicative of rigid DNA. Their findings indicated that DNA sequences with low intrinsic cyclizability tend to exhibit a high predicted ProT. The PsgM04F phage genome exhibits a ProT of −7.57 compared to the −8.21 from the PsgM02F phage genome. The PsgM04F phage genome has, therefore, a higher ProT and a lower average cyclizability value than the PsgM02F phage genome, which might indicate that the PsgM04F phage genome is, in fact, more rigid than the PsgM02F phage genome.

In summary, the PsgM02F phage genome exhibits a higher cyclizability, suggesting greater dsDNA flexibility compared to that of the PsgM04F phage genome. However, this conclusion does not imply rigidity in the PsgM04F phage genome; as discussed earlier, specific dinucleotides in dsDNA contribute to its flexibility. However, the over-representation of certain dinucleotides and the influence of DNA shape suggest that the PsgM02F phage genome may be more suitable for bending than the PsgM04F phage genome.

The phage dsDNA translocation process upon infection of a susceptible bacterial host cell involves dynamic interactions between translocating machinery and dsDNA [129]. The cyclizability of DNA can impact the structural dynamics during this process. A DNA molecule with higher cyclizability, such as the PsgM02F phage genome, may positively influence the structural dynamics of DNA during dsDNA translocation.

Hence, a less flexible phage genome (as was the case for phage PsgM04F) apparently leads to a lower virion morphogenesis yield (12 virions per host cell, in the case of phage PsgM04F) upon successful infection of a susceptible bacterial host cell and in turn, a more flexible (bendable) phage genome (as was the case for phage PsgM02F) apparently leads to a higher virion morphogenesis yield (124 virions per host cell, in the case of phage PsgM02F). These conclusions are fully backed up by findings from Basu et al. [27] and later confirmed by Balcão et al. [30], that dsDNA sequences rich in the dinucleotide TA display much higher intrinsic cyclizability values, which is consistent with the deployed mechanistic rationale that the bacterial host cell RNA-polymerase might interact and negotiate better with a dsDNA sequence endowed with higher intrinsic cyclizability (and therefore more flexible), leading to higher transcription rates with concomitant higher levels of protein synthesis following infection and associated translocation of phage DNA into the bacterial cytoplasm upon contraction of the phage tail sheath.

The similarities and relationships between phages PsgM02F and PsgM04F and other related prokaryotic dsDNA viruses were analyzed using the web-based ViPTree program (https://www.genome.jp/viptree (accessed on 6 January 2024)) [78,79], resulting in the viral proteomic trees displayed in Figure 14. The results obtained showed clearly that phage PsgM02F was grouped into one small group with 10 other phages (Figure 14a2), with nearly all such phages belonging to the *Straboviridae* family according to the updated ICTV taxonomic classification, whereas phage PsgM04F was grouped into one small group with other 19 phages (Figure 14b2).

According to a recent work by Smug et al. [130], biological modularity enhances evolutionary adaptability, with phages displaying extensive genomic modularity [130]. Maybe, just maybe, the mechanical features of a phage genome change according to the changes in its modularity. No correlation between the phages’ structural (genomic mechanical) features and their host range could be established. Since the host range of a phage is directly linked to its ability to recognize and attach to receptors on the surface of a susceptible bacterial host cell through appropriate phage protein binding domains (which result, obviously, from protein-coding genes in its genome), with attachment of a phage onto a susceptible host cell being mandatory prior to translocation of its genome into the cell cytoplasm, culminating in infection of the cell, maybe there is a relation between the mechanical features of the phage genome and its ability to attach onto the cell. However, at the moment, with the information already conveyed herein, these are mere speculative hypotheses that will require further and deeper analysis.

## 5. Conclusions

The annotated coding sequences with predicted functions in the genome of phages PsgM02F and PsgM04F did not encode depolymerases, toxins, virulence factors, antibiotic resistance, or integrase enzymes with typical structural proteins such as capsid, tail, baseplate, and spike proteins together with DNA metabolism-related proteins being annotated in the two-phage genomes. Hence, a strictly lytic lifestyle can safely be assumed for both phages, making them adequate for Psg control trials. The results presented herein clearly suggest that different phage DNA shapes influence their mechanical (flexibility) properties, which, in turn, might play an effective role in the virion morphogenesis yield upon translocation of phage DNA into the bacterial cytoplasm following contraction of the virion tail sheath. Hence, a more flexible (bendable) genome appears therefore to favor the production of phage proteins in large numbers within infected bacterial cells with concomitant higher (mature) virion morphogenesis yields.

## Figures and Tables

**Table 1:** Approximate dimensions of phages PsgM02F and PsgM04F virions (average measurements of 7 virions).

Structural Feature	Phage Virion Dimensions	
	Phage PsgM02F	Phage PsgM04F
Capsid length (nm)	101.3 ± 4.0	77.5 ± 3.5
Capsid width (nm)	81.5 ± 2.8	73.0 ± 2.2
Tail length (nm)	108.7 ± 0.8	102.5 ± 4.3
Tail thickness (nm)	21.9 ± 3.0	15.6 ± 3.8

**Table 2:** Genomic features of phages PsgM02F and PsgM04F genomes.

Feature	Phage PsgM02F	Phage PsgM04F
NCBI/Genbank accession number	OR584013	OR584014
Genome size	165,282 bp	151,205 bp
Number of PE reads mapping in the final assembly	47,224,300 (91.64%)	25,251,477 (99.48%)
Average sequencing coverage (calculated as (length of reads × number of reads mapping)/genome size)	171,636×	33,443×
GC content	35.37%	42.30%
tRNA genes	11	11
Protein-coding genes (CDS) predicted	278	324
• With function assigned	136 (48.9%)	73 (22.5%)
• Hypothetical/unknown function	142 (51.1%)	251 (77.5%)
Similar phage genome sequences NCBI/RefSeq accession number of similar phage	Escherichia phage HP3/Shigella phage Sf21 GCF_002619885.1/GCF_002955385.1	Escherichia phage phiWec190 LC739539.1
Morphotype	*Myovirus*	*Myovirus*
Family/Genus	*Straboviridae*/*Tequatrovirus*	*Stephanstirmvirinae*/*Phapecoctavirus*

**Table 3:** Results from the one-way ANOVA statistical analysis performed on the whole set of predicted intrinsic cyclizability data of phages PsgM02F and PsgM04F genomes.

Source of Variation	SS	df	MS	*F*-Ratio	*p*-Value	*F*-Critical
Between Groups	17.288	1.000	17.288	428.585	9.105 × 10^−95^	3.842
Within Groups	1823.138	45,197	0.040			
Total	1840.426	45,198				

Notes: SS—sum of squares; df—degrees of freedom; MS—mean square (variance estimate; MS = SS/df); *F*-ratio—MS_between_/MS_within_; *p*-value—probability that the mean will be ≥ (or ≤) than observed results, given that the null hypothesis is true; *F*-critical—statistical *F*-value (1; 45,197; 0.05).

## Data Availability

The phage genome sequences described in this work have been deposited in GenBank NCBI (National Center for Biotechnology Information) under accession numbers OR584013 (PsgM02F) and OR584014 (PsgM04F).

## Supplementary Materials

The following supporting information can be downloaded at: https://www.mdpi.com/article/10.3390/genes15010113/s1, Table S1: Annotation of phage PsgM02F genome. Table S2. Annotation of phage PsgM04F genome.
